# Precision Medicine in Myeloid Neoplasia: Challenges and Opportunities

**DOI:** 10.3390/jpm15020049

**Published:** 2025-01-26

**Authors:** Michael J. Hochman, Joshua P. Muniz, Nikolaos Papadantonakis

**Affiliations:** 1 Department of Hematology and Medical Oncology, Winship Cancer Institute of Emory University, Atlanta, GA 30322, USA; 2 Aflac Cancer & Blood Disorders Center, Children’s Healthcare of Atlanta, Atlanta, GA 30329, USA

**Keywords:** myeloid malignancies, small-molecule inhibitors, functional precision medicine, tumor neoantigens

## Abstract

High-risk myeloid neoplasms encompass a group of hematologic malignancies known to cause significant cytopenias, which are accompanied by the risk of end-organ damage. They tend to have an aggressive clinical course and limit life expectancy in the absence of effective treatments. The adoption of precision medicine approaches has been limited by substantive diversity in somatic mutations, limited fraction of patients with targetable genetic lesions, and the prolonged turnaround times of pertinent genetic tests. Efforts to incorporate targeted agents into first-line treatment, rapidly determine pre-treatment molecular or cytogenetic aberrations, and evaluate functional vulnerabilities ex vivo hold promise for advancing the use of precision medicine in these malignancies. Given the relative accessibility of malignant cells from blood and bone marrow, precision medicine strategies hold great potential to shape future standard-of-care approaches to patients with high-risk myeloid malignancies. This review aims to summarize the development of the targeted therapies currently available to treat these blood cancers, most notably acute myeloid leukemia, and also evaluate future opportunities and challenges related to the integration of personalized approaches.

## 1. Introduction

Myeloid lineage blood cells are responsible for the production of many vital blood elements, including erythrocytes, granulocytes, platelets, and monocytes. Malignancies developing from myeloid progenitor and stem cells range in terms of natural history, symptomatology, and the need for treatment. They include relatively ubiquitous diseases such as myelodysplastic syndromes (MDS) and less common ones like primary myelofibrosis (PMF). Regardless of specific diagnosis, most high-risk myeloid neoplasms (HRMNs) share aggressive clinical courses, resulting in limited life expectancy in the absence of treatments, including potentially curative allogeneic hematopoietic stem cell transplants (allo-SCT). Unfortunately, the intensive cytotoxic therapies traditionally used for HRMNs, specifically acute myeloid leukemia (AML), have limited efficacy [1], cause substantial treatment-related mortality [2], are highly cost ineffective [3], and may not be feasible for all patients given the predilection for these malignancies in older, comorbid populations [4].

The development of well-tolerated drugs that selectively target neoplastic over non-malignant cells has become a highly coveted feature in all of oncology, but especially in the treatment of patients with HRMNs, for whom limited therapeutic options previously existed. While B-cell lymphoid neoplasms have led to the development of a number of highly effective drugs specific to B-cell lineage cell surface antigens and monitoring technologies very specific to malignant clones [5], such precision medicine approaches have been more gradually developed and are less robust in myeloid malignancies. With the discovery of the Philadelphia chromosome [6,7] and the development of the BCR-ABL tyrosine kinase inhibitor (TKI) imatinib in chronic myeloid leukemia (CML) at the turn of the twenty-first century [8], precision oncologic approaches were realized in the treatment of HRMNs. This transformed certain malignancies (CML, most famously) from life-threatening entities to chronic diseases.

**Figure 1:**
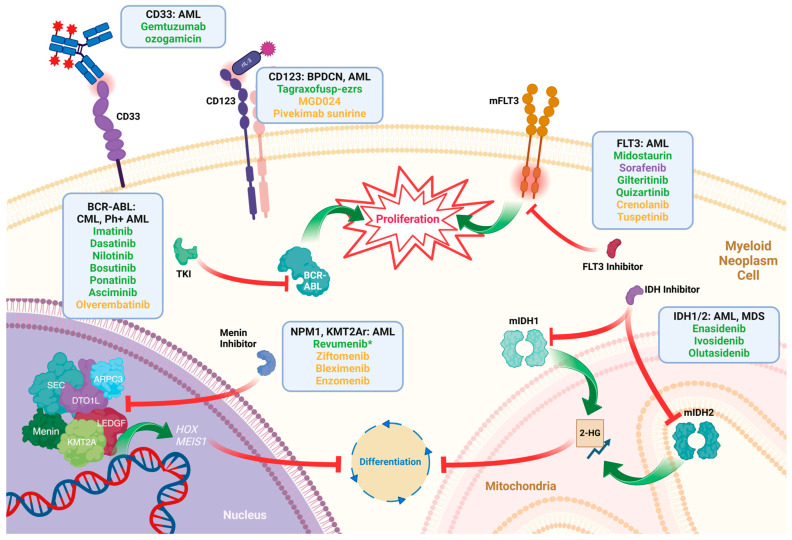
A selection of drugs currently approved and under study for treating HRMNs. Listed are the drug targets and the myeloid malignancies for which these drugs are used. Drugs in green are already approved, those in yellow are under study, and purple drugs are used off-label. 2-HG: 2-hydroxyglutarate; AML: acute myeloid leukemia; BPDCN: blastic plasmacytoid dendritic cell neoplasm; KMT2Ar: KMT2A-rearranged; MDS: myelodysplastic syndromes; Ph+: Philadelphia chromosome-positive; TKI: tyrosine kinase inhibitor. * FDA-approved for relapsed/refractory *KMT2A*-rearranged AML as of November 2024 and currently in clinical trials for relapsed/refractory *NPM1*-mutant AML.

Ongoing barriers to precision oncologic drug development in HRMNs include few myeloid-specific antigen targets, the risks of on-target off-tumor toxicities, and substantive diversity of somatic mutations [9,10], both between patients with the same malignancy and within the tumor cell population of any given patient. This genetic heterogeneity increases the risk of selecting treatment-resistant or target-negative subclones following therapeutic exposure with targeted agents, as can be seen with development of *BCR-ABL1* kinase domain mutations in CML [11]. Despite these challenges, a multitude of targets relatively specific for myeloid neoplasms have been identified, resulting in a plethora of precision therapeutics being developed in the last 24 years (Figure 1). While such advances are critical, disease heterogeneity limits their applicability—only 60% of patients younger than 60 years old with AML have genetic lesions targetable with current therapeutics, and this drops to 44% in those older than 60 years [12].

Ample opportunities exist to further advance the use of precision medicine in HRMNs. Novel combinatorial approaches in disease subsets with multiple targets (i.e., *NPM1*- and *FLT3*-*ITD* mutated AML) may allow for lessened reliance on traditional cytotoxic agents that form the backbone of AML treatments. Further understanding of tumor cell functional vulnerabilities should allow the development of drugs targeting these subsets and functional biomarkers predictive of responses. Multicenter precision medicine trials are permitting the relatively quick accrual of patients with rare disease subsets and provide a key framework for evaluating new precision medicine drugs.

In this review, we discuss targets of interest in myeloid neoplasms, approved and investigational targeted agents, and functional precision medicine approaches in HRMNs. As drugs used in AML treatment account for most precision medicine research in HRMNs, this is the malignancy focused on in this review.

## 2. Targeting Myeloid Surface Markers

### 2.1. CD33

Even before the development of imatinib, the targeting of myeloid surface markers yielded the first precision drug in myeloid malignancies in 2000: the anti-CD33 antibody–drug conjugate (ADC) gemtuzumab ozogamicin (GO). As the transmembrane glycoprotein CD33 is only expressed in myeloid cells and displays increased expression in multiple myeloid malignancies [13,14], it is inherently an attractive therapeutic target. However, as repeatedly demonstrated with precision medicine drugs, “precision” is only so precise. After receiving accelerated approval by the United States Food and Drug Administration (FDA) in 2000 as a salvage therapy for adults with relapsed AML at a dose of 9 mg/m^2^ on days 1 and 14 due to early-phase trials [15], GO was recalled a decade later due to the increased incidence of veno-occlusive disease (VOD) [16] and hepatotoxicity.

The re-evaluation of GO with lower fractionated dosing (3 mg/m^2^ on days 1, 4, and 7 in combination with induction chemotherapy) in the phase III AFLA-0701 trial showed favorable outcomes among adult patients with de novo AML, particularly event-free survival (EFS is defined as the time from randomization to leukemic relapse, death from any cause, or failure to achieve remission) [17]. This study and others [18,19] informed the reapproval of GO by the FDA in 2017 as a frontline agent that can be combined with intensive induction chemotherapy, with doses at 6 mg/m^2^ recognized to have greater toxicity without greater therapeutic benefit compared to doses of 3 mg/m^2^ [20]. Patients with favorable risk cytogenetics (i.e., core-binding factor AML) had a marked survival benefit per MRC AML15 (5-year overall survival [OS] of 79% with GO versus 51% without) [18], favoring the clinical use of this drug in this population. Further studies found that myeloblast CD33 expression correlates with responses to GO in both pediatric [21] and adult populations [22], though higher doses of GO (i.e., 6 mg/m^2^) may restore therapeutic effects in those with lower CD33 levels [23]. Resistance mechanisms are not completely elucidated and may include CD33 polymorphisms/variants [24], Bcl-2 overexpression, or enhanced payload efflux [25].

### 2.2. CD123

The next surface marker relevant to targeted therapeutics in HRMNs is the alpha chain of the interleukin-3 receptor (IL-3Rα), also known as CD123. While highly expressed on myeloid progenitors [26], plasmacytoid dendritic cells [27], endothelial cells [28], and leukemic blasts [29], it has minimal expression in normal hematopoietic stem cells [29]. Importantly, CD123 is disproportionately expressed on leukemic blasts and leukemic stem cells [29,30,31], suggesting there is a favorable risk/benefit relationship in the use of CD123-targeted therapeutics. Furthermore, experiments in vitro demonstrate the proliferative effects of IL-3 in AML cells [32], suggesting that the inhibition of this pathway could be exploited therapeutically. Finally, AML with high CD123 expression is associated with worse response rates to induction chemotherapy, decreased relapse-free survival (RFS), and worse OS [30,33].

Despite the theoretical promise in targeting this receptor and the initial studies showing evidence of antileukemic activity in preclinical [31,34,35] and early-phase clinical [36] studies for the humanized anti-CD123 monoclonal antibody talacotuzumab, a phase II/III study did not show any improvement in response rate or survival with the addition of this drug to decitabine treatment compared to the use of decitabine alone [37]. In contrast, the anti-CD123 agent tagraxofusp was approved by the FDA [38] for the treatment of blastic plasmacytoid dendritic cell neoplasms (BPDCNs) following a single-arm study of this agent, which showed a 90% overall response rate (ORR) in previously untreated patients [39]. BPDCNs stem from malignant plasmacytoid dendritic cells and often present with cutaneous involvement but can also infiltrate the bone marrow, spleen, and central nervous system [40]. Unlike ADCs, tagraxofusp consists of a payload conjugated to the ligand of the target: recombinant human IL-3 is bound to the cytotoxic A fragment of diphtheria toxin [41]. The most distinct side effect observed was a capillary leak syndrome that caused 2 deaths among the 47 trial patients [39]. Naturally, there is interest in finding a means to incorporate tagraxofusp into the treatment of AML, including in combination with hypomethylating agents (HMA) and the BCL-2 inhibitor venetoclax (VEN) [42,43].

Interest remains in developing other safe and effective anti-CD123 therapies, including bispecific antibody and chimeric antigen receptor (CAR)-T cell-based approaches [44,45,46]. Flotetuzumab was such a bispecific drug, targeting CD123 and T-cell activator CD3ε. It was designed to stimulate T-cell-directed responses against AML cells. It was found to have promising activity in patients with primary induction failure or early relapse [47]. Its development was discontinued in favor of a second-generation agent MGD024, which can be administered via outpatient infusions and is being evaluated in a phase I study (NCT05362773).

Pivekimab sunirine (previously known as IMGN632) is an ADC comprising a high-affinity anti-CD123 antibody linked to an indolinobenzodiazepine pseudodimer payload. It was designed to limit the exposure of normal marrow cells to excess toxicity, as the payload mechanism involves single-strand DNA breakage as opposed to double-strand breakage [48]. A phase I/II trial in patients with relapsed/refractory AML found an ORR of 21% and a composite complete remission (CR) rate of 17% [49], prompting further study of the drug in upfront combination therapies (NCT04086264). Finally, the use of antibody-linked radionucleotides to efficiently deliver radiation lethal to target cells is a unique strategy that has thus far been explored preclinically with an anti-CD123 ADC linked to astatine-211 [50].

## 3. Targeting Genetic Alterations

### 3.1. BCR-ABL1 Tyrosine Kinase Inhibitors

The hallmark of CML is the presence of translocation between chromosomes 9 and 22 or “t(9;22)”, known as the Philadelphia chromosome. Imatinib was the first tyrosine kinase inhibitor (TKI) to demonstrate potent activity against the oncoprotein BCR-ABL1 [51] that results from this translocation. In the pivotal phase III randomized study of interferon-alfa and cytarabine versus imatinib in newly diagnosed chronic-phase CML (n = 1106), 83.3% of patients in the imatinib group were alive at a median follow-up of 10.9 years [52]. Furthermore, 6.9% of patients on imatinib had disease progression from chronic to accelerated or blast-phase disease compared to 12.8% of patients treated with interferon-alfa, which is significantly more aggressive and challenging to treat than chronic-phase disease [52]. Subsequent discontinuation trials further solidified imatinib as the crown jewel of precision oncology (the Stop Imatinib [STIM1] [53,54], CML8 TWISTER [55], and European Stop Tyrosine Kinase Inhibitor [EURO-SKI] trials [56]). These studies demonstrated that patients in sustained, deep molecular remission following imatinib treatment can safely discontinue TKI, with a sizeable fraction obtaining long-term treatment-free remission.

Imatinib is certainly no “silver bullet” for chronic-phase CML. Prognosis in CML varies based on several clinicopathologic factors, including spleen size, additional chromosomal abnormalities, age, and blood count [57]. Resistance and intolerance to imatinib warranted the development of the second-generation TKIs dasatinib, nilotinib, and bosutinib, which were found to be effective in such patient populations [58,59,60]. These drugs are also able to obtain deep responses such as a major molecular response (MMR—a critical treatment milestone tied to favorable treatment outcomes and defined as a 3-log reduction in BCR-ABL1 transcript level [61]) in a greater proportion of patients than imatinib in the upfront treatment of chronic-phase disease, though without any clear benefit in OS [62,63,64]. Strategies to combine imatinib with other drugs in order to modulate immune response are in development [65].

Resistance to second-generation TKIs, and in particular the T315I kinase domain mutation, led to the development of ponatinib and asciminib. Ponatinib is a third-generation TKI that can obtain favorable responses in the majority of patients, with chronic-phase CML [66], whereas asciminib eschews binding to the ATP binding site of BCR-ABL1 (where the other TKIs bind) for the myristoyl site in order to lock the BCR-ABL1 protein in an inactive conformation [67]. Asciminib was more effective than bosutinib in multiply refractory CML [68] and permitted the consideration of combination approaches [69]. Resistance to asciminib and ponatinib prompted the study of the TKI olverembatinib, with promising results seen in the phase Ib study [70].

### 3.2. FLT3 Inhibitors

The treatment of AML with mutations involving the FMS-like tyrosine kinase 3 (FLT3) represents an important advance in precision oncology. While only about one-third of patients with AML have mutations involving *FLT3* (either internal tandem duplications [ITD] or those involving the tyrosine kinase domain [TKD]), the natural history of this disease is usually more aggressive [71,72]. The first FDA-approved agent was midostaurin, a multikinase inhibitor with activity against other receptor tyrosine kinases such as KIT [73]. When combined with intensive chemotherapy in patients with either *FLT3-ITD* or *TKD* mutations, it significantly improved the 4-year OS relative to placebos (51% versus 44%) in the phase III RATIFY trial [74,75].

Subsequently, the FLT3 inhibitor quizartinib was developed to target *FLT3-ITD* and was tested in both the upfront and relapsed AML settings [76,77]. In the upfront setting, the results of the phase III QuANTUM-First study similarly showed improved survival (32 months with quizartinib versus 15 months with placebo) [77], but presented similar rates of composite CR. Though QuANTUM-First permitted the enrollment of patients up to the age of 75 years (as opposed to RATIFY, which required patients to be less than 60 years of age), patients in the 60–75 age range did not appear to receive the same survival benefits from quizartinib in post hoc analysis [77]. Notably, the non-randomized phase 2 trial performed by the German–Austrian Acute Myeloid Leukemia Study Group (AMLSG), incorporating midostaurin and intensive chemotherapy, included patients up to the age of 70 [78]. With the caveat of the historical control as comparator, both younger and older AML patients benefited from midostaurin. Finally, the FLT3 inhibitor crenolanib appeared to have similar beneficial effects for *FLT3*-mutant AML when combined with intensive chemotherapy in upfront treatment [79].

The development of an optimal strategy for the treatment of older patients with *FLT3*-mutant AML is an area of active investigation, given the less robust data for the use of an FLT3 inhibitor in patients older than 60 years receiving intensive chemotherapy. Furthermore, the standard of care for older or comorbid patients with AML is HMA/VEN, for which outcomes in *FLT3*-mutant disease are less favorable [80]. Attempts to use a triplet with a FLT3 inhibitor with VEN and either an HMA or low-dose cytarabine can be effective but expose patients to risks from excessive myelosuppression [81]. Such toxicity can potentially be mitigated by truncating the duration of venetoclax, using FLT3 inhibitors with shorter half-lives like midostaurin, and sequencing the FLT3 inhibitor after chemotherapy [82,83]. Ultimately, larger randomized trials will be needed to cement the role of FLT3 inhibitors in the initial treatment of older or comorbid patients with *FLT3*-mutant AML.

Patients with relapsed/refractory *FLT3*-mutant AML were studied in the ADMIRAL trial, which compared the use of the FLT3 inhibitor gilteritinib to treatment with salvage chemotherapy [84,85]. The majority of patients on trial had relapsed disease (60%), 13% had exposure to a prior FLT3 inhibitor, and approximately 20% underwent prior allo-SCT. The median OS values were 9 months versus 5 months in the gilteritinib and control arm, respectively. The use of gilteritinib was also extended to the post-allo-SCT maintenance setting to select patients with detectable measurable residual disease (MRD) peri-allo-SCT as per the results of the MORPHO study [86].

### 3.3. IDH Inhibitors

Isocitrate dehydrogenase (IDH) mutations impact both the *IDH1* and *IDH2* genes and are reported in AML, MDS, and PMF. They result in neomorphic enzyme activity that results in the conversion of alpha-ketoglutarate into the oncometabolite 2-hydroxyglutarate [87]. *IDH* mutations are quite rare (4%) in PMF cases and are associated with poor outcomes, including leukemic transformation [88]. The incidence of mutations is more prevalent in AML patients (approximately 10% when the pediatric population is included) [89] compared to MDS patients. The mutations are also more prevalent in elderly patients [89].

Enasidenib was the first IDH inhibitor approved, targeting *IDH2* mutations that occur in up to 19% of AML cases [89]. In the first-in-human phase I/II study evaluating this drug in relapsed/refractory patients with AML, it garnered an ORR of 40.3%, a median response duration of 5.8 months, and a median OS of 9.3 months [90]. A unique side effect observed in 6% of patients was differentiation syndrome, characterized by rapid increase in white blood cell count with fever and edema but no actual infection [90]. This drug was the first in non-promyelocytic AML to feature such a differentiating effect, though others followed. 

Two IDH inhibitors were developed to target mutant *IDH1*, namely, ivosidenib and olutasidenib. Ivosidenib was evaluated as single agent in newly diagnosed patients with an *IDH1* mutation who were ineligible to receive standard chemotherapy [91]. The ORR was 54% and 77% of patients remained in CR at one year. Ivosidenib was also evaluated in the phase III AGILE trial combined with hypomethylating agent azacitidine (AZA) in patients with newly diagnosed *IDH1*-mutated AML, compared to the use of AZA and placebo [92]. The median OS of the AZA and ivosidenib arm was 29 months, and this was over three times that of the AZA and placebo arm [92,93]. Finally, ivosidenib gained approval for relapsed/refractory *IDH1*-mutant MDS based on a phase I study (AG120-C-001) due to the resolution of transfusion dependence in a significant fraction of patients and attainment of CR in 7 of 18 relapsed/refractory MDS patients [94].

The use of olutasidenib, a relatively newer drug, resulted in durable remission in patients with relapsed/refractory *IDH1*-mutated AML [95] and appeared to be effective in combination with AZA [96]. Patients with relapsed/refractory AML post-VEN exposure notably had a poor prognosis, but one small study (n = 18) showed these patients attained relatively favorable outcomes (43.8% composite CR, median response duration not reached) after being treated with olutasidenib [97].

### 3.4. Menin Inhibitors 

Rearrangements of the lysine methyltransferase 2A (*KMT2A*) gene and mutations in nucleophosmin 1 (*NPM1*) are two of the most common genetic alterations seen in AML, occurring at rates of about 10% and 30%, respectively [98]. In the last 10 years, multiple preclinical and phase I/II studies showed the promise of menin inhibitors in *NPM1*- and *KMT2A*-rearranged AML. Menin is a nuclear protein coded by the gene *MEN1*, which is located on chromosome 11q13 [98,99]. Menin acts as a scaffolding protein that interacts with cell signaling and gene regulators. In AML, menin interacts with *KMT2A* to promote leukemogenesis by inducing aberrant stem cell gene expression through the activation of homeobox (*HOX*) genes and its cofactor MEIS1 [99]. Mutated *NPM1*- and *KMT2A*-rearranged proteins both require interactions with menin to form the protein complex necessary to block normal myeloid cell differentiation and to promote leukemogenesis [98,99]. Therefore, menin inhibitors are under investigation as small-molecule inhibitors in AML patients with *NPM1* mutations and *KMT2A* rearrangements.

Preclinical studies in mouse models showed that menin inhibition led to the reversal of aberrant gene expression and the eventual differentiation of leukemic cells or apoptosis [100,101]. These studies suggest that the aberrant menin–*KMT2A* complex leads to myeloid progenitor cell self-renewal and proliferation, which in time leads to the development of leukemia [100,102,103]. In knock-in mouse models, blocking menin prevented the development of leukemia [102]. Patient-derived xenograft models of these AML subtypes also showed that menin inhibition led to remission or the regression of disease [101,104].

The success of preclinical studies led to the development of multiple menin inhibitors for use in early-phase studies. The first in-human study with the menin inhibitor SNDX-5613, now revumenib, evaluated this drug in patients with relapsed/refractory acute leukemia in a phase I/II AUGMENT 101 study [105]. Patients were heavily pre-treated with a median of 4 prior therapies and 38.9% underwent allo-SCT (n = 14). The overall response rate was 63.2% (n = 36), with 10 (18%) achieving CR [106]. A sizeable number of patients treated with revumenib achieved MRD negativity by multiparametric flow cytometry (15 of 22 patients with composite CR) [105,107]. In addition, a proportion of patients were able to proceed to allo-SCT after achieving a response [105,106,108]. Revumenib was approved by the FDA for relapsed/refractory *KMT2A*-rearranged AML in November 2024.

Another menin inhibitor, KO-539 (also known as ziftomenib), is currently under study in the phase II KOMET-001 trial. One-quarter of patients (n = 9) with *KMT2A* rearrangement or *NPM1* mutation have achieved a composite CR thus far [109]; study enrollment is ongoing. Interestingly, due to more frequent and severe differentiation syndrome in patients with *KMT2A*-rearranged disease, the enrollment of this group was stopped [109]. Only one patient developed a resistance mutation to menin inhibition (*MEN1* M327I) [109], compared to the nearly 40% of patients who developed one with revumenib [110].

### 3.5. Other Pathways

The targeting of KIT and the JAK-STAT pathway have proven effective in the treatment of systemic mastocytosis and PMF with KIT inhibitors and JAK inhibitors, respectively [111,112], but are outside the scope of this review. A summary of multiple approved precision medicine agents in HRMNs, as discussed above, is shown in Table 1.

## 4. Opportunities in Precision Approaches for HRMNs

### 4.1. Novel Antigen Targets 

Given the poor outcomes seen in many HRMNs despite the advances detailed above, aggressive research efforts are required to further expand both potential treatment targets and therapies by using novel precision approaches. One area of particular interest is to target tumor cell neoantigens using antibody or adoptive cellular therapies. For instance, mutations in *CALR* drive about one-fifth of myeloproliferative neoplasms (MPNs) [120]. Mutant calreticulin proteins are aberrantly exposed on the MPN cell surface while being bound to the thrombopoietin receptor MPL, causing the constitutive stimulation of the JAK-STAT pathway in MPN cells but not in *CALR* wildtype hematopoietic cells. This makes it an ideal precision medicine target [120,121,122]. The anti-calreticulin monoclonal antibody INCA033989 has demonstrated early promise with this approach, as preclinical studies demonstrate that it attenuates JAK-STAT signaling and decreases the burden of *CALR*-mutated hematopoietic stem and progenitor cells to the exclusion of *CALR* wildtype cells [123].

Few myeloid neoplasms display uniform surface expression of fundamentally dependent oncoproteins, as with *CALR*-mutant MPNs; thus, much interest lies in targeting other neoantigens [124]. The presence of several genes recurrently mutated in myeloid malignancies, such as *U2AF1* [125], *IDH2*^R140Q^, *IDH1*^R132H^, *FLT3*^D835Y^, and *DNMT3A*^R882H^ [126], results in immunogenic neoantigens that appear targetable by clonal T-cell receptor (TCR)-based cellular therapies. Wilms’ tumor 1 (WT1) may be a broader target of interest as it is overexpressed in the majority of patients with AML and MDS, and also to significantly lesser degrees in healthy tissue [127,128,129,130]. Further, clinical studies indicate that WT1-specific TCR-targeting T-cell therapies are safe [131] and hold promise as a post allo-SCT consolidation tool [132]. Nonetheless, TCR-based adoptive cellular therapy is human leukocyte antigen (HLA)-restricted [133], creating increased complexity in the scaling and delivery of such therapies in practice. 

CAR-T cell therapies circumvent the HLA-restricted antigen expression seen in the treatment of hematologic malignancies. These have proliferated quite successfully in the treatment of lymphoid malignancies such as B-cell acute lymphoblastic leukemia (B-ALL) [134] and multiple myeloma [135]. However, CAR-T-based therapies have not progressed in the treatment of myeloid malignancies to such a degree owing to a multitude of factors [136], including significant on-target off-tumor toxicities [137,138] and challenges with T-cell dysfunction in HRMNs [139,140,141]. The targeting of myeloid antigens that are more specific for malignant clones in combination [142] using inventive CAR-T platforms [142,143,144,145] may ultimately render CAR-T therapies safe and effective in HRMNs. Lastly, Crispr/Cas9 has been used to engineer CD33-negative hematopoietic stem and progenitor cells, allowing for the use of anti-CD33 therapies (including CAR-T cells) without the myeloablative toxicities seen in the absence of this gene editing [146].

### 4.2. Novel Genetic Targets

While drugs targeting activating mutations like *FLT3* and alterations key to leukemogenesis (e.g., menin and aberrant IDH enzymes) offer important progress in precision approaches to AML, the mutations more often found in MDS or chronic myelomonocytic leukemia (CMML) are ripe for targeting. Spliceosome mutations, such as *SRSF2*, *U2AF1*, and *ZRSR2*, are adverse prognostic features typically associated with secondary AML [147] and are relatively common in myeloid neoplasms [148,149], making the spliceosome an attractive drug target. Furthermore, the mutual exclusivity observed among spliceosome mutations suggests that the complete dysfunction of spliceosome machinery could be synthetically lethal [150,151,152], providing an opportunity for spliceosome inhibitors to be used in a setting with such mutations. Drugs with various mechanisms of spliceosome inhibition are under development and in trials [153].

Further insights into myeloid neoplasm biology will likely provide other potential targets. Nicotinamide phosphoribosyltransferase (NAMPT), a rate-limiting enzyme in the nicotinamide adenine dinucleotide (NAD) salvage pathway, is a potential target in cancer [154] and of particular interest in myeloid neoplasms due to the location of the *NAMPT* gene at 7q22.3. Monosomy 7 and deletion 7q are highly adverse cytogenetic features [155] that result in *NAMPT* haploinsufficiency. This greater reliance on NAMPT makes AML cells particularly vulnerable to NAMPT inhibition in preclinical models [156], potentially offering a means to target this particularly high-risk form of AML.

Finally, concerted efforts to target high-risk genetic lesions have not been successful outside of the development of menin inhibitors for *KMT2A* rearrangements. Specifically, *TP53*-mutant AML and MDS are notorious for having poor therapeutic responses and survival [157,158,159]. Eprenetapopt was specifically designed as a refolding agent for the mutant p53 protein, being able to restore the ability of *TP53*-mutant myeloid neoplasm cells to undergo apoptosis [160]. Early-phase studies demonstrated promising responses [161,162,163], but ultimately phase III studies did not meet their primary endpoint [164]. Similarly, there was significant excitement regarding the use of magrolimab, an anti-CD47 monoclonal antibody thought to promote tumor cell phagocytosis by macrophages due to the blockade of an immune checkpoint [165]. Despite the perception that this drug was notably active in *TP53*-mutant MDS and AML, its phase III study was discontinued due to futility [166,167]. As a drug targeting *TP53* mutation is an area of tremendous clinical need, further study of TP53 biology is likely essential to develop more effective approaches.

### 4.3. Functional Precision Medicine Targets

Aside from the targeting of cell surface markers with relatively greater expression on malignant myeloid cells or enzymatic pathways that are aberrant in subsets of myeloid malignancies, precision approaches can be used to develop drugs that target metabolic or epigenetic vulnerabilities. Arguably, the most successful drug used to target such an abnormality is VEN, based on preclinical data demonstrating BCL-2 expression is associated with chemoresistance and excess BCL-2 dependence permits resistance to apoptosis [168,169,170]. The initial study of VEN as a salvage monotherapy showed limited activity [171], but combination with AZA was remarkable in an early-phase trial [172]. Given the use of AZA monotherapy in older (i.e., age 75 years and older) and frail patients with AML ineligible for intensive chemotherapy, the promise of adding VEN to AZA for this same population prompted a randomized phase III trial, dubbed VIALE-A [173]. This confirmed the superiority of AZA/VEN over AZA in terms of remission rate and survival, with subsequent studies suggesting the addition of VEN is less valuable in patients with *TP53*-mutant disease [174,175]. As the indication for the use of VEN (in combination with AZA) is based on patient-related factors, not measures of BCL-2 dependence, in practicality, it is used as a backbone therapy in AML as opposed to a precision drug.

Not all drugs developed to address functional issues panned out in HRMNs. Tamibarotene, an agonist to the retinoic acid receptor alpha (RARα), was piloted in a study of MDS and AML via the identification of functional dependency on RARα through transcriptomic analysis [176]. Data from early-phase studies of tamibarotene in combination with AZA in AML patients with RARα overexpression appeared positive [177], but trials of tamibarotene in both higher-risk MDS and AML were later terminated due to the lack of benefit found in subsequent analyses. 

Recently, the identification of an *FLT3* mutation-like gene expression profile in 25–50% of *FLT3*-wildtype cases suggested there may be a subset of AML patients that may respond to FLT3 inhibition [178]. Indeed, early-phase studies of quizartinib in relapsed/refractory AML showed responses in about one-third of *FLT3*-wildtype patients evaluated [179]. Though midostaurin has not been found to be effective in combination with intensive chemotherapy for *FLT3*-wildtype disease [180], the results from the phase II QUIWI study suggested improved survival in patients with *FLT3*-wildtype AML undergoing intensive induction who were randomized to quizartinib over a placebo [181]. The findings appear strongest in the *FLT3*-like group [182], and further study will evaluate the use of quizartinib in this setting in the international phase III QuANTUM-Wild study (NCT06578247).

## 5. Challenges and Limitations to Precision Medicine in Myeloid Neoplasms

Precision medicine drugs in HRMNs are not without side effects, which largely occur due to the effect of drugs on pathways or markers shared by both neoplastic cells and healthy ones. TKI toxicities in the setting of CML are common and may require dose adjustments [183]. In QuANTUM-First, approximately one-third of patients treated with quizartinib had a QT interval of more than 450 msec (after correcting with Fridericia’s formula) compared to 17% in placebo arm, and grade 3/4 prolongation occurred in 3% of quizartinib-treated patients [77]. An important cause of the toxicity of IDH inhibitors, gilteritinib, and menin inhibitors is differentiation syndrome, which can be life-threatening and may be difficult to promptly recognize [184,185]

While drugs like gilteritinib or revumenib offer clear benefits in a heavily pre-treated, relapsed/refractory setting [84,106], only about one-fifth of patients obtain CRs with count recovery and no patients are cured without subsequent allo-SCT. Resistance can be on-target (related to mutations in the drug target) or secondary to emerging mutations (as in the development of *RAS* mutations in patients treated with FLT3 inhibitors) [110,186,187]. The bone marrow microenvironment, recognized itself as abnormal in HRMNs [188,189], may not permit precision drugs to be effective and is perhaps a therapeutic target itself [190].

With the incorporation of precision drugs into the upfront setting in combination with “backbone” chemotherapies, as occurs with midostaurin (paired with cytarabine and anthracycline-based intensive induction) and ivosidenib (paired with HMA), outcomes appear more durable. This is likely owing to the heterogeneity of AML within any given patient and the decreased ability for resistant subclones to develop. The challenge of using precision diagnostics to allocate targeted drugs upfront to patients with HRMN is discussed in more detail below. 

## 6. Translating Personalized Approaches to Patients

**Figure 2:**
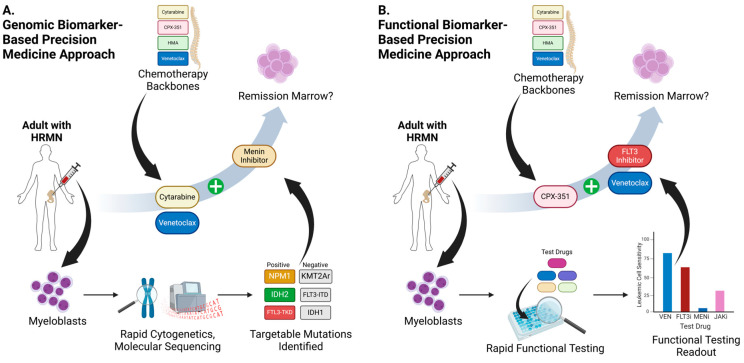
Precision-based approaches to clinical trials in HRMNs using genomic biomarkers (**A**) and functional biomarkers (**B**). FLT3i: FLT3 inhibitor; HMA: hypomethylating agent; HRMN: high-risk myeloid neoplasm; JAKi: JAK inhibitor; MENi: menin inhibitor; VEN: venetoclax.

Although we have discussed a plethora of drugs that can target genetic lesions, abnormal surface markers, or functional abnormalities specific to AML and other HRMNs, the identification of the optimal drugs and drug combination for any given patient has some practical barriers that has resulted in most targeted agents being relegated for the relapsed and refractory HRMN population. Though the use of rapid cytogenetics is feasible [191], molecular genetics laboratories are less often equipped to obtain detailed next-generation sequencing data with a sufficiently fast turnaround time that allows for incorporation of this genetic information into the frontline setting. Additionally, patients with AML are not always stable enough to delay treatment for 2–3 weeks, resulting in treatment being chosen based on other clinicopathologic factors. Another way of circumventing this is to rapidly ascertain these genetic characteristics and assign treatment based on them by using an optimized workflow in a genomic biomarker-based precision medicine approach (Figure 2A). The first iteration of this approach is the BEAT AML trial, which consists of a Master protocol in which clinicians can obtain next-generation sequencing (NGS) data from FoundationOne within a 7-day period [192]. Subsequent treatment assignment is then performed for a sub-study based on the initial characterization of a patient’s AML genetic makeup. This approach was found to be feasible and safe for patients enrolled [192], as seen with some of the BEAT sub-studies readouts [193].

Similarly, the National Cancer Institute’s Myeloid Malignancies Molecular Analysis for Therapy Choice (“MyeloMATCH”) study hopes to take a rigorous precision-based approach for patients, evaluating patients within a Master Screening and Reassessment Protocol (MSRP) that returns comprehensive genomic data within 72 h and permits patients to pursue a targeted trial during any phase of their treatment (e.g., induction, for MRD, or post-SCT) as applicable. Patients treated for newly diagnosed AML or MDS are stratified into “Older Adult”, “Younger Adult”, and “MDS” baskets to further personalize studies for them [194,195,196]. Though the use of rapid genomic sequencing to drive these precision medicine trials is exciting, such technologies and precision approaches are largely not applicable to resource-limited medical settings. A laudable goal of MyeloMATCH is to have its trials open at community hospitals in order to expand treatment access to traditionally underserved populations [196].

An alternative approach to the development of genomic-based biomarkers is centered around the use of functional biomarkers to assign treatment (Figure 2B). The accessibility of tumor cells in most HRMNs makes such an approach attractive. Essentially, this approach exposes myeloid cancer cells to a panel of drugs ex vivo for a limited duration, and then assesses for evidence of drug sensitivity or signs of apoptosis. Treatment is then assigned based on the results of this test. This approach has been enacted using different technologies, such as image-based single-cell functional precision testing (pharmacoscopy) [197]. It was evaluated in multiple aggressive hematologic malignancies in the EXALT trial [198] and SMARTrial [199], and was assessed in AML patients in the DARTT-1 trial [200]. Such trials demonstrated the feasibility of this approach, but whether cell visualization is the most optimal means to assess ex vivo responses remains to be seen. 

## 7. Conclusions

With greater elucidation of the genetic alterations in HRMNs, precision medicine has become more prominent in myeloid malignancies; in fact, most drug approvals in this space have largely been those designed to target surface markers or HRMN-specific genetic alterations. Menin inhibitors represent an important advance that is relevant to the most common genetic alteration in AML, *NPM1* mutations. Large, multicenter trials allowing rapid acquisition of genomic data will allow more patients to be evaluated using precision-based approaches with upfront therapy, marking a significant advance in the care of AML. Significant barriers remain to the adaptation of precision oncology approaches for all patients with HRMNs; ideally, all patients require at least one targetable lesion and these agents should often be combined in upfront treatments. Functional precision medicine technologies may allow for treatment personalization beyond the genomic-based biomarkers currently used to assign targeted drugs. Given the advances in precision oncology in HRMNs over the last two decades, the ability to customize and personalize therapy for patients with myeloid malignancies may soon be within reach.

## Figures and Tables

**Table 1:** Summary table describing FDA-approved precision medicine drugs for high-risk myeloid neoplasms, their targets, and clinical uses. AML: acute myeloid leukemia. AZA: azacitidine; BMT: bone marrow transplantation; BP: blast phase; BPDCN: blastic plasmacytoid dendritic cell neoplasm; CBF: core-binding factor; CML: chronic myeloid leukemia; CP: chronic phase; CR: complete response; DS: differentiation syndrome; EFS: event-free survival; ITD: internal tandem duplication; IVO: ivosidenib; MDS: myelodysplastic syndromes; MMR: major molecular response; Mo: months; MRD: measurable residual disease; Mut: mutated; ND: newly diagnosed; ORR: overall response rate; OS: overall survival; REMS: Risk Evaluation and Mitigation Strategy; R/R: relapsed/refractory; SM: systemic mastocytosis; TKD: tyrosine kinase domain; TKI: tyrosine kinase inhibitor; URI: upper respiratory infection; VOD: veno-occlusive disease. * originally FDA-approved in 2000, GO was voluntarily withdrawn from the market in 2010 due to trials that demonstrated safety concerns and did not confirm clinical benefit. It was then reapproved in 2017. † initially approved in December 2012, ponatinib was briefly voluntarily withdrawn due to serious safety concerns including the risk of arterial occlusive events. It was then reapproved in December 2020. ‡ initially approved in 2021 for chronic-phase CML following the failure of two or more tyrosine kinase inhibitors or following the development of the T315I mutation, asciminib was then granted accelerated approval for the treatment of newly diagnosed chronic-phase CML in 2024.

Drug Name	Molecular Target(s)	Myeloid Neoplasm(s)	FDA Approval	Year(s) Approved	Major Studies	Trial Outcomes	Clinical Uses	Key Toxicities
Gemtuzumab Ozogamicin (GO)	CD33	AML	CD33+ AML, ND (adults) and R/R (adults, pediatrics)	2017 *	AFLA-0701 [17] MRC AML15 [18] COG AAML0531 [113] NCRI AML17 [20]	Median EFS (17.3 mo vs. 9.5 mo) OS at 5 years: 79% vs. 51% (favorable risk cytogenetics)	Induction for ND CBF AML, CD33+ Cytoreduction for CD33+ or extramedullary AML (off-label) [114]	Hepatotoxicity (including VOD), infusion reactions, cytopenias
Tagraxofusp	CD123	BPDCN	CD123+ BPDCN (patients ≥ 2 years old)	2018	Pemmaraju et al. [39]	ORR: 90% (previously untreated patients)	ND or R/R BPDCN AML (currently in phase II trials)	Capillary leak syndrome, fever, weight gain
Ponatinib	BCR-ABL1 (ATP-binding pocket)	CML	Adults with R/R CML to 2 or more prior TKIs, or detected T315I mutation	2020 †	PACE [66] OPTIC [115]	MMR: 40% at any time	R/R CP-CML (following at least 2 prior lines) CML with T315I mutation; BP-CML (off-label)	Arterial occlusive events, pancreatitis, hyperlipidemia, congestive heart failure, cytopenias
Asciminib	BCR-ABL1 (myristoyl-binding pocket)	CML	CP-CML, frontline or R/R	2021; 2024 ‡	ASCEMBL [68] CABL001X2101 [116] ASC4FIRST [117]	MMR: 25% (R/R) and 42% (T315I) at 24 wks; 68% at 48 wks (ND)	CP-CML, ND and R/R CML-BP (off-label)	URIs, pancreatitis, hypertriglyceridemia
Midostaurin	FLT3 (ITD or TKD), KIT	AML, SM	Adults with ND *FLT3*-mut AML receiving intensive chemotherapy; adults with SM (both *KIT*-mut and wildtype)	2017	AML: CALGB10603 (RATIFY) [74] SM: Gotlib et al. [118]	OS at 4 years (*FLT3*-mut AML): 63.7% vs. 55.7% (placebo)	Used in induction and consolidation Frontline monotherapy in advanced SM	Febrile neutropenia, mucositis, musculoskeletal pain, hyperglycemia, gastrointestinal toxicities
Gilteritinib	FLT3 (ITD or TKD)	AML	Adults with R/R *FLT3*-mut AML	2018	ADMIRAL [84] MORPHO [86]	Median OS: 9 mo vs. 5 mo (salvage chemotherapy)	Used as salvage monotherapy Post-BMT maintenance if +FLT3 MRD peri-BMT	Myalgia/arthralgia, elevated transaminases, pancreatitis, dyspnea, diarrhea, QT prolongation, DS
Quizartinib	FLT3 (ITD only)	AML	Adults with ND *FLT3*-*ITD* AML receiving intensive chemotherapy	2023	QuANTUM-First [77] QuANTUM-R [76]	Median OS: 32 mo vs. 15 mo (placebo)	Used in induction, consolidation, and as maintenance (not post-BMT); salvage monotherapy (off-label)	QT prolongation, cardiac arrest, torsades de pointes (REMS program), hepatotoxicity, cytopenias
Enasidenib	IDH2	AML	Adults with R/R *IDH2*-mut AML	2017	AG221-C-001 [90]	ORR: 40.3%; complete remission in 19.3%	Salvage monotherapy	DS, diarrhea, hyperbilirubinemia, anorexia
Ivosidenib	IDH1	AML, MDS	Adults with *IDH1*-mut ND AML not fit for intensive chemotherapy or R/R *IDH1*-mut AML; *IDH1*-mut R/R MDS	2022 (AML), 2023 (MDS)	AG120-C-001 [119]; AGILE [92]	ORR: 41.6%; complete remission in 21.6% (R/R AML); median OS 24 mo AZA/IVO vs. 8 mo AZA; ORR (MDS): 83.3%	Frontline *IDH1*-mut AML with AZA; salvage monotherapy for either AML or MDS	DS, gastrointestinal toxicities, rash, QT prolongation
Olutasidenib	IDH1	AML	Adults with R/R *IDH1*-mut AML	2022	Study 2102-HEM-101 [95]	ORR: 48%	Salvage monotherapy	Elevated transaminases, DS, dyspnea, fevers, rash
Revumenib	Menin	AML	Patients ≥ 1 years old with R/R *KMT2A*-Rearranged AML	2024	SNDX-5613-0700 (AUGMENT-101) [106]	ORR: 63.2%, 17.5% in complete remission; median OS 8 mo	Salvage monotherapy in clinical trials for *NPM1*-mut AML	DS, QT prolongation, musculoskeletal pain, elevated transaminases

## Data Availability

No new data were created or analyzed in this study.

## References

[1] Rajakumaraswamy N., Gandhi M., Wei A.H., Sallman D.A., Daver N.G., Mo S., Iqbal S., Karalliyadda R., Chen M., Wang Y. (2024). Real-World Effectiveness of Azacitidine in Treatment-Naive Patients with Higher-Risk Myelodysplastic Syndromes. Clin. Lymphoma Myeloma Leuk..

[2] Appelbaum F.R., Gundacker H., Head D.R., Slovak M.L., Willman C.L., Godwin J.E., Anderson J.E., Petersdorf S.H. (2006). Age and Acute Myeloid Leukemia. Blood.

[3] Menzin J., Lang K., Earle C.C., Kerney D., Mallick R. (2002). The Outcomes and Costs of Acute Myeloid Leukemia Among the Elderly. Arch. Intern. Med..

[4] Sorror M.L., Gooley T.A., Storer B.E., Gerds A.T., Sekeres M.A., Medeiros B.C., Wang E.S., Shami P.J., Adekola K., Luger S. (2023). An 8-Year Pragmatic Observation Evaluation of the Benefits of Allogeneic HCT in Older and Medically Infirm Patients with AML. Blood.

[5] Ching T., Duncan M.E., Newman-Eerkes T., McWhorter M.M.E., Tracy J.M., Steen M.S., Brown R.P., Venkatasubbarao S., Akers N.K., Vignali M. (2020). Analytical Evaluation of the clonoSEQ Assay for Establishing Measurable (Minimal) Residual Disease in Acute Lymphoblastic Leukemia, Chronic Lymphocytic Leukemia, and Multiple Myeloma. BMC Cancer.

[6] Rowley J.D. (1973). Letter: A New Consistent Chromosomal Abnormality in Chronic Myelogenous Leukaemia Identified by Quinacrine Fluorescence and Giemsa Staining. Nature.

[7] Bartram C.R., de Klein A., Hagemeijer A., van Agthoven T., Geurts van Kessel A., Bootsma D., Grosveld G., Ferguson-Smith M.A., Davies T., Stone M. (1983). Translocation of C-Ab1 Oncogene Correlates with the Presence of a Philadelphia Chromosome in Chronic Myelocytic Leukaemia. Nature.

[8] O’Brien S.G., Guilhot F., Larson R.A., Gathmann I., Baccarani M., Cervantes F., Cornelissen J.J., Fischer T., Hochhaus A., Hughes T. (2003). Imatinib Compared with Interferon and Low-Dose Cytarabine for Newly Diagnosed Chronic-Phase Chronic Myeloid Leukemia. N. Engl. J. Med..

[9] DeZern A.E. (2018). Treatments Targeting MDS Genetics: A Fool’s Errand?. Hematol. Am. Soc. Hematol. Educ. Program.

[10] Awada H., Durmaz A., Gurnari C., Kishtagari A., Meggendorfer M., Kerr C.M., Kuzmanovic T., Durrani J., Shreve J., Nagata Y. (2021). Machine Learning Integrates Genomic Signatures for Subclassification beyond Primary and Secondary Acute Myeloid Leukemia. Blood.

[11] Gorre M.E., Mohammed M., Ellwood K., Hsu N., Paquette R., Rao P.N., Sawyers C.L. (2001). Clinical Resistance to STI-571 Cancer Therapy Caused by BCR-ABL Gene Mutation or Amplification. Science.

[12] Bataller A., DiNardo C.D., Bazinet A., Daver N.G., Maiti A., Borthakur G., Short N., Sasaki K., Jabbour E.J., Issa G.C. (2024). Targetable Genetic Abnormalities in Patients with Acute Myeloblastic Leukemia across Age Groups. Am. J. Hematol..

[13] Taussig D.C., Pearce D.J., Simpson C., Rohatiner A.Z., Lister T.A., Kelly G., Luongo J.L., Danet-Desnoyers G.-A.H., Bonnet D. (2005). Hematopoietic Stem Cells Express Multiple Myeloid Markers: Implications for the Origin and Targeted Therapy of Acute Myeloid Leukemia. Blood.

[14] Jilani I., Estey E., Huh Y., Joe Y., Manshouri T., Yared M., Giles F., Kantarjian H., Cortes J., Thomas D. (2002). Differences in CD33 Intensity between Various Myeloid Neoplasms. Am. J. Clin. Pathol..

[15] Larson R.A., Sievers E.L., Stadtmauer E.A., Löwenberg B., Estey E.H., Dombret H., Theobald M., Voliotis D., Bennett J.M., Richie M. (2005). Final Report of the Efficacy and Safety of Gemtuzumab Ozogamicin (Mylotarg) in Patients with CD33-Positive Acute Myeloid Leukemia in First Recurrence. Cancer.

[16] McKoy J.M., Angelotta C., Bennett C.L., Tallman M.S., Wadleigh M., Evens A.M., Kuzel T.M., Trifilio S.M., Raisch D.W., Kell J. (2007). Gemtuzumab Ozogamicin-Associated Sinusoidal Obstructive Syndrome (SOS): An Overview from the Research on Adverse Drug Events and Reports (RADAR) Project. Leuk. Res..

[17] Lambert J., Pautas C., Terré C., Raffoux E., Turlure P., Caillot D., Legrand O., Thomas X., Gardin C., Gogat-Marchant K. (2019). Gemtuzumab Ozogamicin for de Novo Acute Myeloid Leukemia: Final Efficacy and Safety Updates from the Open-Label, Phase III ALFA-0701 Trial. Haematologica.

[18] Burnett A.K., Hills R.K., Milligan D., Kjeldsen L., Kell J., Russell N.H., Yin J.A.L., Hunter A., Goldstone A.H., Wheatley K. (2011). Identification of Patients with Acute Myeloblastic Leukemia Who Benefit from the Addition of Gemtuzumab Ozogamicin: Results of the MRC AML15 Trial. J. Clin. Oncol..

[19] Delaunay J., Recher C., Pigneux A., Witz F., Vey N., Blanchet O., Lefebvre P., Luquet I., Guillerme I., Volteau C. (2011). Addition of Gemtuzumab Ozogamycin to Chemotherapy Improves Event-Free Survival but Not Overall Survival of AML Patients with Intermediate Cytogenetics Not Eligible for Allogeneic Transplantation. Results of the GOELAMS AML 2006 IR Study. Blood.

[20] Burnett A., Cavenagh J., Russell N., Hills R., Kell J., Jones G., Nielsen O.J., Khwaja A., Thomas I., Clark R. (2016). Defining the Dose of Gemtuzumab Ozogamicin in Combination with Induction Chemotherapy in Acute Myeloid Leukemia: A Comparison of 3 Mg/M2 with 6 Mg/M2 in the NCRI AML17 Trial. Haematologica.

[21] Pollard J.A., Loken M., Gerbing R.B., Raimondi S.C., Hirsch B.A., Aplenc R., Bernstein I.D., Gamis A.S., Alonzo T.A., Meshinchi S. (2016). CD33 Expression and Its Association With Gemtuzumab Ozogamicin Response: Results From the Randomized Phase III Children’s Oncology Group Trial AAML0531. JCO.

[22] Olombel G., Guerin E., Guy J., Perrot J.-Y., Dumezy F., de Labarthe A., Bastie J.-N., Legrand O., Raffoux E., Plesa A. (2016). The Level of Blast CD33 Expression Positively Impacts the Effect of Gemtuzumab Ozogamicin in Patients with Acute Myeloid Leukemia. Blood.

[23] Khan N., Hills R.K., Virgo P., Couzens S., Clark N., Gilkes A., Richardson P., Knapper S., Grimwade D., Russell N.H. (2017). Expression of CD33 Is a Predictive Factor for Effect of Gemtuzumab Ozogamicin at Different Doses in Adult Acute Myeloid Leukaemia. Leukemia.

[24] Teich K., Krzykalla J., Kapp-Schwoerer S., Gaidzik V.I., Schlenk R.F., Paschka P., Weber D., Fiedler W., Kühn M.W.M., Schroeder T. (2021). Cluster of Differentiation 33 Single Nucleotide Polymorphism Rs12459419 Is a Predictive Factor in Patients with Nucleophosmin1-Mutated Acute Myeloid Leukemia Receiving Gemtuzumab Ozogamicin. Haematologica.

[25] Khoury R., Saleh K., Khalife N., Saleh M., Chahine C., Ibrahim R., Lecesne A. (2023). Mechanisms of Resistance to Antibody-Drug Conjugates. Int. J. Mol. Sci..

[26] Emerson S.G., Yang Y.C., Clark S.C., Long M.W. (1988). Human Recombinant Granulocyte-Macrophage Colony Stimulating Factor and Interleukin 3 Have Overlapping but Distinct Hematopoietic Activities. J. Clin. Investig..

[27] MacDonald K.P.A., Munster D.J., Clark G.J., Dzionek A., Schmitz J., Hart D.N.J. (2002). Characterization of Human Blood Dendritic Cell Subsets. Blood.

[28] Korpelainen E.I., Gamble J.R., Vadas M.A., Lopez A.F. (1996). IL-3 Receptor Expression, Regulation and Function in Cells of the Vasculature. Immunol. Cell Biol..

[29] Jordan C.T., Upchurch D., Szilvassy S.J., Guzman M.L., Howard D.S., Pettigrew A.L., Meyerrose T., Rossi R., Grimes B., Rizzieri D.A. (2000). The Interleukin-3 Receptor Alpha Chain Is a Unique Marker for Human Acute Myelogenous Leukemia Stem Cells. Leukemia.

[30] Graf M., Hecht K., Reif S., Pelka-Fleischer R., Pfister K., Schmetzer H. (2004). Expression and Prognostic Value of Hemopoietic Cytokine Receptors in Acute Myeloid Leukemia (AML): Implications for Future Therapeutical Strategies. Eur. J. Haematol..

[31] Jin L., Lee E.M., Ramshaw H.S., Busfield S.J., Peoppl A.G., Wilkinson L., Guthridge M.A., Thomas D., Barry E.F., Boyd A. (2009). Monoclonal Antibody-Mediated Targeting of CD123, IL-3 Receptor Alpha Chain, Eliminates Human Acute Myeloid Leukemic Stem Cells. Cell Stem Cell.

[32] Vellenga E., Ostapovicz D., O’Rourke B., Griffin J.D. (1987). Effects of Recombinant IL-3, GM-CSF, and G-CSF on Proliferation of Leukemic Clonogenic Cells in Short-Term and Long-Term Cultures. Leukemia.

[33] Testa U., Riccioni R., Militi S., Coccia E., Stellacci E., Samoggia P., Latagliata R., Mariani G., Rossini A., Battistini A. (2002). Elevated Expression of IL-3Ralpha in Acute Myelogenous Leukemia Is Associated with Enhanced Blast Proliferation, Increased Cellularity, and Poor Prognosis. Blood.

[34] Lee E.M., Yee D., Busfield S.J., McManus J.F., Cummings N., Vairo G., Wei A., Ramshaw H.S., Powell J.A., Lopez A.F. (2015). Efficacy of an Fc-Modified Anti-CD123 Antibody (CSL362) Combined with Chemotherapy in Xenograft Models of Acute Myelogenous Leukemia in Immunodeficient Mice. Haematologica.

[35] Xie L.H., Biondo M., Busfield S.J., Arruda A., Yang X., Vairo G., Minden M.D. (2017). CD123 Target Validation and Preclinical Evaluation of ADCC Activity of Anti-CD123 Antibody CSL362 in Combination with NKs from AML Patients in Remission. Blood Cancer J..

[36] Smith B.D., Roboz G.J., Walter R.B., Altman J.K., Ferguson A., Curcio T.J., Orlowski K.F., Garrett L., Busfield S.J., Barnden M. (2014). First-in Man, Phase 1 Study of CSL362 (Anti-IL3Rα/Anti-CD123 Monoclonal Antibody) in Patients with CD123+ Acute Myeloid Leukemia (AML) in CR at High Risk for Early Relapse. Blood.

[37] Montesinos P., Roboz G.J., Bulabois C.-E., Subklewe M., Platzbecker U., Ofran Y., Papayannidis C., Wierzbowska A., Shin H.J., Doronin V. (2021). Safety and Efficacy of Talacotuzumab plus Decitabine or Decitabine Alone in Patients with Acute Myeloid Leukemia Not Eligible for Chemotherapy: Results from a Multicenter, Randomized, Phase 2/3 Study. Leukemia.

[38] Food and Drug Administration (2019). FDA Approves Tagraxofusp-Erzs for Blastic Plasmacytoid Dendritic Cell Neoplasm.

[39] Pemmaraju N., Lane A.A., Sweet K.L., Stein A.S., Vasu S., Blum W., Rizzieri D.A., Wang E.S., Duvic M., Sloan J.M. (2019). Tagraxofusp in Blastic Plasmacytoid Dendritic-Cell Neoplasm. N. Engl. J. Med..

[40] Jain A., Sweet K. (2023). Blastic Plasmacytoid Dendritic Cell Neoplasm. J. Natl. Compr. Cancer Netw..

[41] Frankel A., Liu J.-S., Rizzieri D., Hogge D. (2008). Phase I Clinical Study of Diphtheria Toxin-Interleukin 3 Fusion Protein in Patients with Acute Myeloid Leukemia and Myelodysplasia. Leuk. Lymphoma.

[42] Lane A.A., Garcia J.S., Raulston E.G., Garzon J.L., Galinsky I., Baxter E.W., Leonard R., DeAngelo D.J., Luskin M.R., Reilly C.R. (2024). Phase 1b Trial of Tagraxofusp in Combination with Azacitidine with or without Venetoclax in Acute Myeloid Leukemia. Blood Adv..

[43] Green S., Parkin B., Lai C., Gojo I., Zeidner J. (2023). AML-274 TAGALONG Trial: Phase II Study of Tagraxofusp and Azacitidine With or Without Venetoclax in Newly Diagnosed Secondary AML after Previous Exposure to Hypomethylating Agents. Clin. Lymphoma Myeloma Leuk..

[44] Watts J., Lin T.L., Mims A., Patel P., Lee C., Shahidzadeh A., Shami P., Cull E., Cogle C.R., Wang E. (2021). Post-Hoc Analysis of Pharmacodynamics and Single-Agent Activity of CD3xCD123 Bispecific Antibody APVO436 in Relapsed/Refractory AML and MDS Resistant to HMA or Venetoclax Plus HMA. Front. Oncol..

[45] Cai T., Gouble A., Black K.L., Skwarska A., Naqvi A.S., Taylor D., Zhao M., Yuan Q., Sugita M., Zhang Q. (2022). Targeting CD123 in Blastic Plasmacytoid Dendritic Cell Neoplasm Using Allogeneic Anti-CD123 CAR T Cells. Nat. Commun..

[46] Aldoss I., Clark M., Song J.Y., Pullarkat V. (2020). Targeting the Alpha Subunit of IL-3 Receptor (CD123) in Patients with Acute Leukemia. Hum. Vaccines Immunother..

[47] Uy G.L., Aldoss I., Foster M.C., Sayre P.H., Wieduwilt M.J., Advani A.S., Godwin J.E., Arellano M.L., Sweet K.L., Emadi A. (2021). Flotetuzumab as Salvage Immunotherapy for Refractory Acute Myeloid Leukemia. Blood.

[48] Kovtun Y., Jones G.E., Adams S., Harvey L., Audette C.A., Wilhelm A., Bai C., Rui L., Laleau R., Liu F. (2018). A CD123-Targeting Antibody-Drug Conjugate, IMGN632, Designed to Eradicate AML While Sparing Normal Bone Marrow Cells. Blood Adv..

[49] Daver N.G., Montesinos P., DeAngelo D.J., Wang E.S., Papadantonakis N., Todisco E., Sweet K.L., Pemmaraju N., Lane A.A., Torres-Miñana L. (2024). Pivekimab Sunirine (IMGN632), a Novel CD123-Targeting Antibody–Drug Conjugate, in Relapsed or Refractory Acute Myeloid Leukaemia: A Phase 1/2 Study. Lancet Oncol..

[50] Laszlo G.S., Orozco J.J., Kehret A.R., Lunn M.C., Huo J., Hamlin D.K., Scott Wilbur D., Dexter S.L., Comstock M.L., O’Steen S. (2022). Development of [211At]Astatine-Based Anti-CD123 Radioimmunotherapy for Acute Leukemias and Other CD123+ Malignancies. Leukemia.

[51] Kantarjian H., Sawyers C., Hochhaus A., Guilhot F., Schiffer C., Gambacorti-Passerini C., Niederwieser D., Resta D., Capdeville R., Zoellner U. (2002). Hematologic and Cytogenetic Responses to Imatinib Mesylate in Chronic Myelogenous Leukemia. N. Engl. J. Med..

[52] Hochhaus A., Larson R.A., Guilhot F., Radich J.P., Branford S., Hughes T.P., Baccarani M., Deininger M.W., Cervantes F., Fujihara S. (2017). Long-Term Outcomes of Imatinib Treatment for Chronic Myeloid Leukemia. N. Engl. J. Med..

[53] Mahon F.-X., Réa D., Guilhot J., Guilhot F., Huguet F., Nicolini F., Legros L., Charbonnier A., Guerci A., Varet B. (2010). Discontinuation of Imatinib in Patients with Chronic Myeloid Leukaemia Who Have Maintained Complete Molecular Remission for at Least 2 Years: The Prospective, Multicentre Stop Imatinib (STIM) Trial. Lancet Oncol..

[54] Etienne G., Guilhot J., Rea D., Rigal-Huguet F., Nicolini F., Charbonnier A., Guerci-Bresler A., Legros L., Varet B., Gardembas M. (2017). Long-Term Follow-Up of the French Stop Imatinib (STIM1) Study in Patients With Chronic Myeloid Leukemia. J. Clin. Oncol..

[55] Ross D.M., Branford S., Seymour J.F., Schwarer A.P., Arthur C., Yeung D.T., Dang P., Goyne J.M., Slader C., Filshie R.J. (2013). Safety and Efficacy of Imatinib Cessation for CML Patients with Stable Undetectable Minimal Residual Disease: Results from the TWISTER Study. Blood.

[56] Mahon F.-X., Pfirrmann M., Dulucq S., Hochhaus A., Panayiotidis P., Almeida A., Mayer J., Hjorth-Hansen H., Janssen J.J.W.M., Mustjoki S. (2024). European Stop Tyrosine Kinase Inhibitor Trial (EURO-SKI) in Chronic Myeloid Leukemia: Final Analysis and Novel Prognostic Factors for Treatment-Free Remission. JCO.

[57] Iezza M., Cortesi S., Ottaviani E., Mancini M., Venturi C., Monaldi C., De Santis S., Testoni N., Soverini S., Rosti G. (2023). Prognosis in Chronic Myeloid Leukemia: Baseline Factors, Dynamic Risk Assessment and Novel Insights. Cells.

[58] Talpaz M., Shah N.P., Kantarjian H., Donato N., Nicoll J., Paquette R., Cortes J., O’Brien S., Nicaise C., Bleickardt E. (2006). Dasatinib in Imatinib-Resistant Philadelphia Chromosome-Positive Leukemias. N. Engl. J. Med..

[59] Kantarjian H., Giles F., Wunderle L., Bhalla K., O’Brien S., Wassmann B., Tanaka C., Manley P., Rae P., Mietlowski W. (2006). Nilotinib in Imatinib-Resistant CML and Philadelphia Chromosome-Positive ALL. N. Engl. J. Med..

[60] Gambacorti-Passerini C., Cortes J.E., Lipton J.H., Kantarjian H.M., Kim D.-W., Schafhausen P., Crescenzo R., Bardy-Bouxin N., Shapiro M., Noonan K. (2018). Safety and Efficacy of Second-Line Bosutinib for Chronic Phase Chronic Myeloid Leukemia over a Five-Year Period: Final Results of a Phase I/II Study. Haematologica.

[61] Cortes J., Quintás-Cardama A., Kantarjian H.M. (2011). Monitoring Molecular Response in Chronic Myeloid Leukemia. Cancer.

[62] Cortes J.E., Saglio G., Kantarjian H.M., Baccarani M., Mayer J., Boqué C., Shah N.P., Chuah C., Casanova L., Bradley-Garelik B. (2016). Final 5-Year Study Results of DASISION: The Dasatinib Versus Imatinib Study in Treatment-Naïve Chronic Myeloid Leukemia Patients Trial. JCO.

[63] Kantarjian H.M., Hughes T.P., Larson R.A., Kim D.-W., Issaragrisil S., le Coutre P., Etienne G., Boquimpani C., Pasquini R., Clark R.E. (2021). Long-Term Outcomes with Frontline Nilotinib versus Imatinib in Newly Diagnosed Chronic Myeloid Leukemia in Chronic Phase: ENESTnd 10-Year Analysis. Leukemia.

[64] Cortes J.E., Gambacorti-Passerini C., Deininger M.W., Mauro M.J., Chuah C., Kim D.-W., Dyagil I., Glushko N., Milojkovic D., le Coutre P. (2018). Bosutinib Versus Imatinib for Newly Diagnosed Chronic Myeloid Leukemia: Results From the Randomized BFORE Trial. J. Clin. Oncol..

[65] Heibl S., Buxhofer-Ausch V., Schmidt S., Webersinke G., Lion T., Piringer G., Kuehr T., Wolf D., Melchardt T., Greil R. (2020). A Phase 1 Study to Evaluate the Feasibility and Efficacy of the Addition of Ropeginterferon Alpha-2b to Imatinib Treatment in Patients with Chronic Phase Chronic Myeloid Leukemia (CML) Not Achieving a Deep Molecular Response (Molecular Remission 4.5)-AGMT_CML 1. Hematol. Oncol..

[66] Cortes J.E., Kim D.-W., Pinilla-Ibarz J., le Coutre P.D., Paquette R., Chuah C., Nicolini F.E., Apperley J.F., Khoury H.J., Talpaz M. (2018). Ponatinib Efficacy and Safety in Philadelphia Chromosome-Positive Leukemia: Final 5-Year Results of the Phase 2 PACE Trial. Blood.

[67] Wylie A.A., Schoepfer J., Jahnke W., Cowan-Jacob S.W., Loo A., Furet P., Marzinzik A.L., Pelle X., Donovan J., Zhu W. (2017). The Allosteric Inhibitor ABL001 Enables Dual Targeting of BCR-ABL1. Nature.

[68] Réa D., Mauro M.J., Boquimpani C., Minami Y., Lomaia E., Voloshin S., Turkina A., Kim D.-W., Apperley J.F., Abdo A. (2021). A Phase 3, Open-Label, Randomized Study of Asciminib, a STAMP Inhibitor, vs Bosutinib in CML after 2 or More Prior TKIs. Blood.

[69] Eide C.A., Zabriskie M.S., Stevens S.L.S., Antelope O., Vellore N.A., Than H., Schultz A.R., Clair P., Bowler A.D., Pomicter A.D. (2019). Combining the Allosteric Inhibitor Asciminib with Ponatinib Suppresses Emergence of and Restores Efficacy Against Highly Resistant BCR-ABL1 Mutants. Cancer cell.

[70] Jabbour E., Oehler V.G., Koller P.B., Jamy O., Lomaia E., Hunter A.M., Uspenskaya O., Samarina S., Mukherjee S., Cortes J.E. (2024). Olverembatinib After Failure of Tyrosine Kinase Inhibitors, Including Ponatinib or Asciminib: A Phase 1b Randomized Clinical Trial. JAMA Oncol..

[71] Kottaridis P.D., Gale R.E., Frew M.E., Harrison G., Langabeer S.E., Belton A.A., Walker H., Wheatley K., Bowen D.T., Burnett A.K. (2001). The Presence of a FLT3 Internal Tandem Duplication in Patients with Acute Myeloid Leukemia (AML) Adds Important Prognostic Information to Cytogenetic Risk Group and Response to the First Cycle of Chemotherapy: Analysis of 854 Patients from the United Kingdom Medical Research Council AML 10 and 12 Trials. Blood.

[72] Ambinder A.J., Levis M. (2020). Potential Targeting of FLT3 Acute Myeloid Leukemia. Haematologica.

[73] Schlenk R.F., Kayser S. (2018). Midostaurin: A Multiple Tyrosine Kinases Inhibitor in Acute Myeloid Leukemia and Systemic Mastocytosis. Recent Results Cancer Res.

[74] Stone R.M., Mandrekar S.J., Sanford B.L., Laumann K., Geyer S., Bloomfield C.D., Thiede C., Prior T.W., Döhner K., Marcucci G. (2017). Midostaurin plus Chemotherapy for Acute Myeloid Leukemia with a *FLT3* Mutation. N. Engl. J. Med..

[75] Levis M. (2017). Midostaurin Approved for FLT3-Mutated AML. Blood.

[76] Cortes J.E., Khaled S., Martinelli G., Perl A.E., Ganguly S., Russell N., Krämer A., Dombret H., Hogge D., Jonas B.A. (2019). Quizartinib versus Salvage Chemotherapy in Relapsed or Refractory FLT3-ITD Acute Myeloid Leukaemia (QuANTUM-R): A Multicentre, Randomised, Controlled, Open-Label, Phase 3 Trial. Lancet Oncol..

[77] Erba H.P., Montesinos P., Kim H.-J., Patkowska E., Vrhovac R., Žák P., Wang P.-N., Mitov T., Hanyok J., Kamel Y.M. (2023). Quizartinib plus Chemotherapy in Newly Diagnosed Patients with FLT3-Internal-Tandem-Duplication-Positive Acute Myeloid Leukaemia (QuANTUM-First): A Randomised, Double-Blind, Placebo-Controlled, Phase 3 Trial. Lancet.

[78] Döhner H., Weber D., Krzykalla J., Fiedler W., Wulf G., Salih H., Lübbert M., Kühn M.W.M., Schroeder T., Salwender H. (2022). Midostaurin plus Intensive Chemotherapy for Younger and Older Patients with AML and FLT3 Internal Tandem Duplications. Blood Adv..

[79] Wang E.S., Goldberg A.D., Tallman M., Walter R.B., Karanes C., Sandhu K., Vigil C.E., Collins R., Jain V., Stone R.M. (2024). Crenolanib and Intensive Chemotherapy in Adults With Newly Diagnosed FLT3-Mutated AML. JCO.

[80] Bataller A., Bazinet A., DiNardo C.D., Maiti A., Borthakur G., Daver N.G., Short N.J., Jabbour E.J., Issa G.C., Pemmaraju N. (2024). Prognostic Risk Signature in Patients with Acute Myeloid Leukemia Treated with Hypomethylating Agents and Venetoclax. Blood Adv..

[81] Short N.J., Daver N., Dinardo C.D., Kadia T., Nasr L.F., Macaron W., Yilmaz M., Borthakur G., Montalban-Bravo G., Garcia-Manero G. (2024). Azacitidine, Venetoclax, and Gilteritinib in Newly Diagnosed and Relapsed or Refractory *FLT3* -Mutated AML. JCO.

[82] Yilmaz M., Muftuoglu M., DiNardo C.D., Kadia T.M., Konopleva M.Y., Borthakur G., Pemmaraju N., Short N.J., Alvarado Valero Y., Maiti A. (2023). Phase I/II Study of Quizartinib, Venetoclax, and Decitabine Triple Combination in FLT3-ITD Mutated AML. Blood.

[83] Chua C.C., Hsu B., Enjeti A.K., Bajel A., Marlton P., Fleming S., Hiwase D., Ma C.K.K., Browett P.J., Perera T. (2024). A Phase II Randomized Trial Comparing Low-Dose Cytarabine and Venetoclax +/− Midostaurin in Non-Adverse Cytogenetic Risk Acute Myeloid Leukemia: The ALLG AMLM25 Intervene Trial. Blood.

[84] Perl A.E., Martinelli G., Cortes J.E., Neubauer A., Berman E., Paolini S., Montesinos P., Baer M.R., Larson R.A., Ustun C. (2019). Gilteritinib or Chemotherapy for Relapsed or Refractory *FLT3* -Mutated AML. N. Engl. J. Med..

[85] Perl A.E., Larson R.A., Podoltsev N.A., Strickland S., Wang E.S., Atallah E., Schiller G.J., Martinelli G., Neubauer A., Sierra J. (2022). Follow-up of Patients with R/R FLT3-Mutation-Positive AML Treated with Gilteritinib in the Phase 3 ADMIRAL Trial. Blood.

[86] Levis M.J., Hamadani M., Logan B., Jones R.J., Singh A.K., Litzow M., Wingard J.R., Papadopoulos E.B., Perl A.E., Soiffer R.J. (2024). Gilteritinib as Post-Transplant Maintenance for AML With Internal Tandem Duplication Mutation of *FLT3*. JCO.

[87] Ward P.S., Patel J., Wise D.R., Abdel-Wahab O., Bennett B.D., Coller H.A., Cross J.R., Fantin V.R., Hedvat C.V., Perl A.E. (2010). The Common Feature of Leukemia-Associated IDH1 and IDH2 Mutations Is a Neomorphic Enzyme Activity Converting Alpha-Ketoglutarate to 2-Hydroxyglutarate. Cancer Cell.

[88] Tefferi A., Jimma T., Sulai N.H., Lasho T.L., Finke C.M., Knudson R.A., McClure R.F., Pardanani A. (2012). IDH Mutations in Primary Myelofibrosis Predict Leukemic Transformation and Shortened Survival: Clinical Evidence for Leukemogenic Collaboration with JAK2V617F. Leukemia.

[89] Zarnegar-Lumley S., Alonzo T.A., Gerbing R.B., Othus M., Sun Z., Ries R.E., Wang J., Leonti A., Kutny M.A., Ostronoff F. (2023). Characteristics and Prognostic Impact of IDH Mutations in AML: A COG, SWOG, and ECOG Analysis. Blood Adv..

[90] Stein E.M., DiNardo C.D., Pollyea D.A., Fathi A.T., Roboz G.J., Altman J.K., Stone R.M., DeAngelo D.J., Levine R.L., Flinn I.W. (2017). Enasidenib in Mutant IDH2 Relapsed or Refractory Acute Myeloid Leukemia. Blood.

[91] Roboz G.J., DiNardo C.D., Stein E.M., de Botton S., Mims A.S., Prince G.T., Altman J.K., Arellano M.L., Donnellan W., Erba H.P. (2020). Ivosidenib Induces Deep Durable Remissions in Patients with Newly Diagnosed IDH1-Mutant Acute Myeloid Leukemia. Blood.

[92] Montesinos P., Recher C., Vives S., Zarzycka E., Wang J., Bertani G., Heuser M., Calado R.T., Schuh A.C., Yeh S.-P. (2022). Ivosidenib and Azacitidine in IDH1-Mutated Acute Myeloid Leukemia. N. Engl. J. Med..

[93] De Botton S., Montesinos P., Vives Polo S., Zarzycka E., Wang J., Riva M., Heuser M., Calado R.T., Schuh A.C., Yeh S.-P. (2023). Updated Efficacy and Safety Data from the AGILE Study in Patients with Newly Diagnosed Acute Myeloid Leukemia Treated with Ivosidenib + Azacitidine Compared to Placebo + Azacitidine. JCO.

[94] DiNardo C.D., Roboz G.J., Watts J.M., Madanat Y.F., Prince G.T., Baratam P., de Botton S., Stein A., Foran J.M., Arellano M.L. (2024). Final Phase 1 Substudy Results of Ivosidenib for Patients with Mutant IDH1 Relapsed/Refractory Myelodysplastic Syndrome. Blood Adv..

[95] de Botton S., Fenaux P., Yee K., Récher C., Wei A.H., Montesinos P., Taussig D.C., Pigneux A., Braun T., Curti A. (2023). Olutasidenib (FT-2102) Induces Durable Complete Remissions in Patients with Relapsed or Refractory IDH1-Mutated AML. Blood Adv..

[96] Watts J.M., Baer M.R., Yang J., Prebet T., Lee S., Schiller G.J., Dinner S.N., Pigneux A., Montesinos P., Wang E.S. (2023). Olutasidenib Alone or with Azacitidine in IDH1-Mutated Acute Myeloid Leukaemia and Myelodysplastic Syndrome: Phase 1 Results of a Phase 1/2 Trial. Lancet Haematol..

[97] Cortes J., Jonas B.A., Schiller G., Mims A., Roboz G.J., Wei A.H., Montesinos P., Ferrell P.B., Yee K.W., Fenaux P. (2024). Olutasidenib in Post-Venetoclax Patients with Mutant Isocitrate Dehydrogenase 1 (mIDH1) Acute Myeloid Leukemia (AML). Leuk. Lymphoma.

[98] Issa G.C., Ravandi F., DiNardo C.D., Jabbour E., Kantarjian H.M., Andreeff M. (2021). Therapeutic Implications of Menin Inhibition in Acute Leukemias. Leukemia.

[99] Candoni A., Coppola G. (2024). A 2024 Update on Menin Inhibitors. A New Class of Target Agents against KMT2A-Rearranged and NPM1-Mutated Acute Myeloid Leukemia. Hematol. Rep..

[100] Grembecka J., He S., Shi A., Purohit T., Muntean A.G., Sorenson R.J., Showalter H.D., Murai M.J., Belcher A.M., Hartley T. (2012). Menin-MLL Inhibitors Reverse Oncogenic Activity of MLL Fusion Proteins in Leukemia. Nat. Chem. Biol..

[101] Klossowski S., Miao H., Kempinska K., Wu T., Purohit T., Kim E., Linhares B.M., Chen D., Jih G., Perkey E. (2020). Menin Inhibitor MI-3454 Induces Remission in MLL1-Rearranged and NPM1-Mutated Models of Leukemia. J. Clin. Investig..

[102] Uckelmann H.J., Kim S.M., Wong E.M., Hatton C., Giovinazzo H., Gadrey J.Y., Krivtsov A.V., Rücker F.G., Döhner K., McGeehan G.M. (2020). Therapeutic Targeting of Preleukemia Cells in a Mouse Model of *NPM1* Mutant Acute Myeloid Leukemia. Science.

[103] Swaminathan M., Bourgeois W., Armstrong S.A., Wang E.S. (2022). Menin Inhibitors in Acute Myeloid Leukemia-What Does the Future Hold?. Cancer J..

[104] Krivtsov A.V., Evans K., Gadrey J.Y., Eschle B.K., Hatton C., Uckelmann H.J., Ross K.N., Perner F., Olsen S.N., Pritchard T. (2019). A Menin-MLL Inhibitor Induces Specific Chromatin Changes and Eradicates Disease in Models of MLL-Rearranged Leukemia. Cancer Cell.

[105] Issa G.C., Aldoss I., DiPersio J., Cuglievan B., Stone R., Arellano M., Thirman M.J., Patel M.R., Dickens D.S., Shenoy S. (2023). The Menin Inhibitor Revumenib in KMT2A-Rearranged or NPM1-Mutant Leukaemia. Nature.

[106] Issa G.C., Aldoss I., Thirman M.J., DiPersio J., Arellano M., Blachly J.S., Mannis G.N., Perl A., Dickens D.S., McMahon C.M. (2024). Menin Inhibition With Revumenib for *KMT2A*-Rearranged Relapsed or Refractory Acute Leukemia (AUGMENT-101). JCO.

[107] Hussain H., Zaidi S.M.F., Hasan S.M., Jahan A.S., Rangwala B.S., Rangwala H.S., Ali M., Farah A.A. (2024). Revumenib (SNDX-5613): A Promising Menin Inhibitor for the Management of Relapsed and Refractory Acute Myeloid Leukaemia (AML). Ann. Med. Surg..

[108] Aldoss I., Issa G.C., Thirman M.J., DiPersio J., Arellano M., Blachly J.S., Mannis G., Perl A., Dickens D., McMahon C.M. (2023). Revumenib Monotherapy in Patients with Relapsed/Refractory KMT2Ar Acute Leukemias: Efficacy and Safety Results from the Augment-101 Phase 1/2 Study. Blood.

[109] Wang E.S., Issa G.C., Erba H.P., Altman J.K., Montesinos P., DeBotton S., Walter R.B., Pettit K., Savona M.R., Shah M.V. (2024). Ziftomenib in Relapsed or Refractory Acute Myeloid Leukaemia (KOMET-001): A Multicentre, Open-Label, Multi-Cohort, Phase 1 Trial. Lancet Oncol..

[110] Perner F., Stein E.M., Wenge D.V., Singh S., Kim J., Apazidis A., Rahnamoun H., Anand D., Marinaccio C., Hatton C. (2023). MEN1 Mutations Mediate Clinical Resistance to Menin Inhibition. Nature.

[111] Chifotides H.T., Bose P. (2024). SOHO State of the Art Update and Next Questions: Current and Emerging Therapies for Systemic Mastocytosis. Clin. Lymphoma Myeloma Leuk..

[112] Hochman M.J., Vale C.A., Hunter A.M. (2024). SOHO State of the Art Updates and Next Questions|Choosing and Properly Using a JAK Inhibitor in Myelofibrosis. Clin. Lymphoma Myeloma Leuk..

[113] Gamis A.S., Alonzo T.A., Meshinchi S., Sung L., Gerbing R.B., Raimondi S.C., Hirsch B.A., Kahwash S.B., Heerema-McKenney A., Winter L. (2014). Gemtuzumab Ozogamicin in Children and Adolescents with de Novo Acute Myeloid Leukemia Improves Event-Free Survival by Reducing Relapse Risk: Results from the Randomized Phase III Children’s Oncology Group Trial AAML0531. J. Clin. Oncol..

[114] Scarlotta M., Webster J., Newman M., Schulz C., Stokvis K., Ambinder A.J., Jain T., Dalton W.B., Gondek L.P., Prince G.T. (2022). Gemtuzumab Ozogamicin for Cytoreduction in Hyperleukocytosis. Blood.

[115] Cortes J., Apperley J., Lomaia E., Moiraghi B., Undurraga Sutton M., Pavlovsky C., Chuah C., Sacha T., Lipton J.H., Schiffer C.A. (2021). Ponatinib Dose-Ranging Study in Chronic-Phase Chronic Myeloid Leukemia: A Randomized, Open-Label Phase 2 Clinical Trial. Blood.

[116] Hughes T.P., Mauro M.J., Cortes J.E., Minami H., Rea D., DeAngelo D.J., Breccia M., Goh Y.-T., Talpaz M., Hochhaus A. (2019). Asciminib in Chronic Myeloid Leukemia after ABL Kinase Inhibitor Failure. N. Engl. J. Med..

[117] Hochhaus A., Wang J., Kim D.-W., Kim D.D.H., Mayer J., Goh Y.-T., le Coutre P., Takahashi N., Kim I., Etienne G. (2024). Asciminib in Newly Diagnosed Chronic Myeloid Leukemia. N. Engl. J. Med..

[118] Gotlib J., Kluin-Nelemans H.C., George T.I., Akin C., Sotlar K., Hermine O., Awan F.T., Hexner E., Mauro M.J., Sternberg D.W. (2016). Efficacy and Safety of Midostaurin in Advanced Systemic Mastocytosis. N. Engl. J. Med..

[119] DiNardo C.D., Stein E.M., de Botton S., Roboz G.J., Altman J.K., Mims A.S., Swords R., Collins R.H., Mannis G.N., Pollyea D.A. (2018). Durable Remissions with Ivosidenib in IDH1-Mutated Relapsed or Refractory AML. N. Engl. J. Med..

[120] How J., Hobbs G.S., Mullally A. (2019). Mutant Calreticulin in Myeloproliferative Neoplasms. Blood.

[121] Araki M., Yang Y., Masubuchi N., Hironaka Y., Takei H., Morishita S., Mizukami Y., Kan S., Shirane S., Edahiro Y. (2016). Activation of the Thrombopoietin Receptor by Mutant Calreticulin in CALR-Mutant Myeloproliferative Neoplasms. Blood.

[122] Kramer F., Mullally A. (2023). Antibody Targeting of Mutant Calreticulin in Myeloproliferative Neoplasms. J. Cell. Mol. Med..

[123] Reis E.S., Buonpane R., Celik H., Marty C., Lei A., Jobe F., Rupar M., Zhang Y., DiMatteo D., Awdew R. (2024). Selective Targeting of Mutated Calreticulin by the Monoclonal Antibody INCA033989 Inhibits Oncogenic Function of MPN. Blood.

[124] Yarchoan M., Johnson B.A., Lutz E.R., Laheru D.A., Jaffee E.M. (2017). Targeting Neoantigens to Augment Antitumour Immunity. Nat. Rev. Cancer.

[125] Biernacki M.A., Lok J., Black R.G., Foster K.A., Cummings C., Woodward K.B., Monahan T., Oehler V.G., Stirewalt D.L., Wu D. (2023). Discovery of U2AF1 Neoantigens in Myeloid Neoplasms. J. Immunother. Cancer.

[126] Leung W.K., Torres Chavez A.G., French-Kim M., Shafer P., Mamonkin M., Hill L.C., Kuvalekar M., Velazquez Y., Watanabe A., Watanabe N. (2024). Targeting IDH2R140Q and Other Neoantigens in Acute Myeloid Leukemia. Blood.

[127] Miwa H., Beran M., Saunders G.F. (1992). Expression of the Wilms’ Tumor Gene (WT1) in Human Leukemias. Leukemia.

[128] Brieger J., Weidmann E., Fenchel K., Mitrou P.S., Hoelzer D., Bergmann L. (1994). The Expression of the Wilms’ Tumor Gene in Acute Myelocytic Leukemias as a Possible Marker for Leukemic Blast Cells. Leukemia.

[129] Menssen H.D., Renkl H.J., Rodeck U., Maurer J., Notter M., Schwartz S., Reinhardt R., Thiel E. (1995). Presence of Wilms’ Tumor Gene (Wt1) Transcripts and the WT1 Nuclear Protein in the Majority of Human Acute Leukemias. Leukemia.

[130] Inoue K., Ogawa H., Sonoda Y., Kimura T., Sakabe H., Oka Y., Miyake S., Tamaki H., Oji Y., Yamagami T. (1997). Aberrant Overexpression of the Wilms Tumor Gene (WT1) in Human Leukemia. Blood.

[131] Tawara I., Kageyama S., Miyahara Y., Fujiwara H., Nishida T., Akatsuka Y., Ikeda H., Tanimoto K., Terakura S., Murata M. (2017). Safety and Persistence of WT1-Specific T-Cell Receptor Gene−transduced Lymphocytes in Patients with AML and MDS. Blood.

[132] Chapuis A.G., Egan D.N., Bar M., Schmitt T.M., McAfee M.S., Paulson K.G., Voillet V., Gottardo R., Ragnarsson G.B., Bleakley M. (2019). T Cell Receptor Gene Therapy Targeting WT1 Prevents Acute Myeloid Leukemia Relapse Post-Transplant. Nat. Med..

[133] Biernacki M.A., Brault M., Bleakley M. (2019). T-Cell Receptor-Based Immunotherapy for Hematologic Malignancies. Cancer J..

[134] Maude S.L., Frey N., Shaw P.A., Aplenc R., Barrett D.M., Bunin N.J., Chew A., Gonzalez V.E., Zheng Z., Lacey S.F. (2014). Chimeric Antigen Receptor T Cells for Sustained Remissions in Leukemia. N. Engl. J. Med..

[135] Raje N., Berdeja J., Lin Y., Siegel D., Jagannath S., Madduri D., Liedtke M., Rosenblatt J., Maus M.V., Turka A. (2019). Anti-BCMA CAR T-Cell Therapy Bb2121 in Relapsed or Refractory Multiple Myeloma. N. Engl. J. Med..

[136] Atilla E., Benabdellah K. (2023). The Black Hole: CAR T Cell Therapy in AML. Cancers.

[137] Kenderian S.S., Ruella M., Shestova O., Klichinsky M., Aikawa V., Morrissette J.J.D., Scholler J., Song D., Porter D.L., Carroll M. (2015). CD33-Specific Chimeric Antigen Receptor T Cells Exhibit Potent Preclinical Activity against Human Acute Myeloid Leukemia. Leukemia.

[138] Laborda E., Mazagova M., Shao S., Wang X., Quirino H., Woods A., Hampton E., Rodgers D., Kim C., Schultz P. (2017). Development of A Chimeric Antigen Receptor Targeting C-Type Lectin-Like Molecule-1 for Human Acute Myeloid Leukemia. IJMS.

[139] Kenderian S.S., June C.H., Gill S., Fortina P., Londin E., Park J.Y., Kricka L.J. (2017). Generating and Expanding Autologous Chimeric Antigen Receptor T Cells from Patients with Acute Myeloid Leukemia. Acute Myeloid Leukemia.

[140] Knaus H.A., Berglund S., Hackl H., Blackford A.L., Zeidner J.F., Montiel-Esparza R., Mukhopadhyay R., Vanura K., Blazar B.R., Karp J.E. (2018). Signatures of CD8^+^ T Cell Dysfunction in AML Patients and Their Reversibility with Response to Chemotherapy. JCI Insight.

[141] Le Dieu R., Taussig D.C., Ramsay A.G., Mitter R., Miraki-Moud F., Fatah R., Lee A.M., Lister T.A., Gribben J.G. (2009). Peripheral Blood T Cells in Acute Myeloid Leukemia (AML) Patients at Diagnosis Have Abnormal Phenotype and Genotype and Form Defective Immune Synapses with AML Blasts. Blood.

[142] Haubner S., Perna F., Köhnke T., Schmidt C., Berman S., Augsberger C., Schnorfeil F.M., Krupka C., Lichtenegger F.S., Liu X. (2019). Coexpression Profile of Leukemic Stem Cell Markers for Combinatorial Targeted Therapy in AML. Leukemia.

[143] Roybal K.T., Rupp L.J., Morsut L., Walker W.J., McNally K.A., Park J.S., Lim W.A. (2016). Precision Tumor Recognition by T Cells With Combinatorial Antigen-Sensing Circuits. Cell.

[144] Salzer B., Schueller C.M., Zajc C.U., Peters T., Schoeber M.A., Kovacic B., Buri M.C., Lobner E., Dushek O., Huppa J.B. (2020). Engineering AvidCARs for Combinatorial Antigen Recognition and Reversible Control of CAR Function. Nat. Commun..

[145] Nixdorf D., Sponheimer M., Berghammer D., Engert F., Bader U., Philipp N., Kazerani M., Straub T., Rohrbacher L., Wange L. (2023). Adapter CAR T Cells to Counteract T-Cell Exhaustion and Enable Flexible Targeting in AML. Leukemia.

[146] Liu Y., Wang S., Schubert M.-L., Lauk A., Yao H., Blank M.F., Cui C., Janssen M., Schmidt C., Göllner S. (2022). CD33-Directed Immunotherapy with Third-Generation Chimeric Antigen Receptor T Cells and Gemtuzumab Ozogamicin in Intact and CD33-Edited Acute Myeloid Leukemia and Hematopoietic Stem and Progenitor Cells. Int. J. Cancer.

[147] Lindsley R.C., Mar B.G., Mazzola E., Grauman P.V., Shareef S., Allen S.L., Pigneux A., Wetzler M., Stuart R.K., Erba H.P. (2015). Acute Myeloid Leukemia Ontogeny Is Defined by Distinct Somatic Mutations. Blood.

[148] Makishima H., Visconte V., Sakaguchi H., Jankowska A.M., Abu Kar S., Jerez A., Przychodzen B., Bupathi M., Guinta K., Afable M.G. (2012). Mutations in the Spliceosome Machinery, a Novel and Ubiquitous Pathway in Leukemogenesis. Blood.

[149] Haferlach T., Nagata Y., Grossmann V., Okuno Y., Bacher U., Nagae G., Schnittger S., Sanada M., Kon A., Alpermann T. (2014). Landscape of Genetic Lesions in 944 Patients with Myelodysplastic Syndromes. Leukemia.

[150] Adamia S., Haibe-Kains B., Pilarski P.M., Bar-Natan M., Pevzner S., Avet-Loiseau H., Lode L., Verselis S., Fox E.A., Burke J. (2014). A Genome-Wide Aberrant RNA Splicing in Patients with Acute Myeloid Leukemia Identifies Novel Potential Disease Markers and Therapeutic Targets. Clin. Cancer Res..

[151] Boddu P.C., Gupta A.K., Roy R., De La Peña Avalos B., Olazabal-Herrero A., Neuenkirchen N., Zimmer J.T., Chandhok N.S., King D., Nannya Y. (2024). Transcription Elongation Defects Link Oncogenic SF3B1 Mutations to Targetable Alterations in Chromatin Landscape. Mol. Cell.

[152] Lee S.C.-W., North K., Kim E., Jang E., Obeng E., Lu S.X., Liu B., Inoue D., Yoshimi A., Ki M. (2018). Synthetic Lethal and Convergent Biological Effects of Cancer-Associated Spliceosomal Gene Mutations. Cancer Cell.

[153] Boussi L., Biswas J., Abdel-Wahab O., Stein E. (2024). Therapeutic Strategies Targeting Aberrant RNA Splicing in Myeloid Malignancies. Br. J. Haematol..

[154] Xiao Y., Elkins K., Durieux J.K., Lee L., Oeh J., Yang L.X., Liang X., DelNagro C., Tremayne J., Kwong M. (2013). Dependence of Tumor Cell Lines and Patient-Derived Tumors on the NAD Salvage Pathway Renders Them Sensitive to NAMPT Inhibition with GNE-618. Neoplasia.

[155] Papaemmanuil E., Döhner H., Campbell P.J. (2016). Genomic Classification in Acute Myeloid Leukemia. N. Engl. J. Med..

[156] Eldfors S., Saad J., Ikonen N., Malani D., Vähä-Koskela M., Gjertsen B.T., Kontro M., Porkka K., Heckman C.A. (2024). Monosomy 7/Del(7q) Cause Sensitivity to Inhibitors of Nicotinamide Phosphoribosyltransferase in Acute Myeloid Leukemia. Blood Adv..

[157] Bernard E., Nannya Y., Hasserjian R.P., Devlin S.M., Tuechler H., Medina-Martinez J.S., Yoshizato T., Shiozawa Y., Saiki R., Malcovati L. (2020). Implications of TP53 Allelic State for Genome Stability, Clinical Presentation and Outcomes in Myelodysplastic Syndromes. Nat. Med..

[158] Grob T., Al Hinai A.S.A., Sanders M.A., Kavelaars F.G., Rijken M., Gradowska P.L., Biemond B.J., Breems D.A., Maertens J., van Marwijk Kooy M. (2022). Molecular Characterization of Mutant *TP53* Acute Myeloid Leukemia and High-Risk Myelodysplastic Syndrome. Blood.

[159] Daver N.G., Iqbal S., Renard C., Chan R.J., Hasegawa K., Hu H., Tse P., Yan J., Zoratti M.J., Xie F. (2023). Treatment Outcomes for Newly Diagnosed, Treatment-Naïve TP53-Mutated Acute Myeloid Leukemia: A Systematic Review and Meta-Analysis. J. Hematol. Oncol..

[160] Lambert J.M.R., Gorzov P., Veprintsev D.B., Söderqvist M., Segerbäck D., Bergman J., Fersht A.R., Hainaut P., Wiman K.G., Bykov V.J.N. (2009). PRIMA-1 Reactivates Mutant P53 by Covalent Binding to the Core Domain. Cancer Cell.

[161] Maslah N., Salomao N., Drevon L., Verger E., Partouche N., Ly P., Aubin P., Naoui N., Schlageter M.-H., Bally C. (2020). Synergistic Effects of PRIMA-1Met (APR-246) and 5-Azacitidine in TP53-Mutated Myelodysplastic Syndromes and Acute Myeloid Leukemia. Haematologica.

[162] Sallman D.A., DeZern A.E., Garcia-Manero G., Steensma D.P., Roboz G.J., Sekeres M.A., Cluzeau T., Sweet K.L., McLemore A., McGraw K.L. (2021). Eprenetapopt (APR-246) and Azacitidine in TP53-Mutant Myelodysplastic Syndromes. J. Clin. Oncol..

[163] Mishra A., Tamari R., DeZern A.E., Byrne M.T., Gooptu M., Chen Y.-B., Deeg H.J., Sallman D., Gallacher P., Wennborg A. (2022). Eprenetapopt Plus Azacitidine After Allogeneic Hematopoietic Stem-Cell Transplantation for TP53-Mutant Acute Myeloid Leukemia and Myelodysplastic Syndromes. J. Clin. Oncol..

[164] Aprea Therapeutics Announces Results of Primary Endpoint from Phase 3 Trial of Eprenetapopt in TP53 Mutant Myelodysplastic Syndromes (MDS)|Aprea Therapeutics. https://ir.aprea.com/news-releases/news-release-details/aprea-therapeutics-announces-results-primary-endpoint-phase-3/.

[165] Sallman D.A., Al Malki M.M., Asch A.S., Wang E.S., Jurcic J.G., Bradley T.J., Flinn I.W., Pollyea D.A., Kambhampati S., Tanaka T.N. (2023). Magrolimab in Combination with Azacitidine in Patients with Higher-Risk Myelodysplastic Syndromes: Final Results of a Phase Ib Study. JCO.

[166] Gilead Statement on the Discontinuation of Magrolimab Study in AML with TP53 Mutations. https://www.gilead.com/company/company-statements/2023/gilead-statement-on-the-discontinuation-of-magrolimab-study-in-aml-with-tp53-mutations.

[167] Gilead To Discontinue Phase 3 ENHANCE Study of Magrolimab Plus Azacitidine in Higher-Risk MDS. https://www.gilead.com/news-and-press/press-room/press-releases/2023/7/gilead-to-discontinue-phase-3-enhance-study-of-magrolimab-plus-azacitidine-in-higher-risk-mds.

[168] Campos L., Rouault J.P., Sabido O., Oriol P., Roubi N., Vasselon C., Archimbaud E., Magaud J.P., Guyotat D. (1993). High Expression of Bcl-2 Protein in Acute Myeloid Leukemia Cells Is Associated with Poor Response to Chemotherapy. Blood.

[169] Vo T.-T., Ryan J., Carrasco R., Neuberg D., Rossi D.J., Stone R.M., Deangelo D.J., Frattini M.G., Letai A. (2012). Relative Mitochondrial Priming of Myeloblasts and Normal HSCs Determines Chemotherapeutic Success in AML. Cell.

[170] Mehta S.V., Shukla S.N., Vora H.H. (2013). Overexpression of Bcl2 Protein Predicts Chemoresistance in Acute Myeloid Leukemia: Its Correlation with FLT3. Neoplasma.

[171] Konopleva M., Pollyea D.A., Potluri J., Chyla B., Hogdal L., Busman T., McKeegan E., Salem A.H., Zhu M., Ricker J.L. (2016). Efficacy and Biological Correlates of Response in a Phase II Study of Venetoclax Monotherapy in Patients with Acute Myelogenous Leukemia. Cancer Discov..

[172] DiNardo C.D., Pratz K.W., Letai A., Jonas B.A., Wei A.H., Thirman M., Arellano M., Frattini M.G., Kantarjian H., Popovic R. (2018). Safety and Preliminary Efficacy of Venetoclax with Decitabine or Azacitidine in Elderly Patients with Previously Untreated Acute Myeloid Leukaemia: A Non-Randomised, Open-Label, Phase 1b Study. Lancet Oncol..

[173] DiNardo C.D., Jonas B.A., Pullarkat V., Thirman M.J., Garcia J.S., Wei A.H., Konopleva M., Döhner H., Letai A., Fenaux P. (2020). Azacitidine and Venetoclax in Previously Untreated Acute Myeloid Leukemia. N. Engl. J. Med..

[174] Pollyea D.A., Pratz K.W., Wei A.H., Pullarkat V., Jonas B.A., Recher C., Babu S., Schuh A.C., Dail M., Sun Y. (2022). Outcomes in Patients with Poor-Risk Cytogenetics with or without TP53 Mutations Treated with Venetoclax and Azacitidine. Clin. Cancer Res..

[175] Badar T., Nanaa A., Atallah E., Shallis R.M., de Camargo Correia Guilherme S., Goldberg A.D., Saliba A.N., Patel A., Bewersdorf J.P., DuVall A.S. (2024). Comparing Venetoclax in Combination with Hypomethylating Agents to Hypomethylating Agent-Based Therapies for Treatment Naive TP53-Mutated Acute Myeloid Leukemia: Results from the Consortium on Myeloid Malignancies and Neoplastic Diseases (COMMAND). Blood Cancer J..

[176] McKeown M.R., Corces M.R., Eaton M.L., Fiore C., Lee E., Lopez J.T., Chen M.W., Smith D., Chan S.M., Koenig J.L. (2017). Superenhancer Analysis Defines Novel Epigenomic Subtypes of Non-APL AML, Including an RARα Dependency Targetable by SY-1425, a Potent and Selective RARα Agonist. Cancer Discov..

[177] de Botton S., Cluzeau T., Vigil C., Cook R.J., Rousselot P., Rizzieri D.A., Liesveld J.L., Fenaux P., Braun T., Banos A. (2023). Targeting RARA Overexpression with Tamibarotene, a Potent and Selective RARα Agonist, Is a Novel Approach in AML. Blood Adv..

[178] Mosquera Orgueira A., Peleteiro Raíndo A., Cid López M., Antelo Rodríguez B., Díaz Arias J.Á., Ferreiro Ferro R., Alonso Vence N., Bendaña López Á., Abuín Blanco A., Bao Pérez L. (2021). Gene Expression Profiling Identifies FLT3 Mutation-like Cases in Wild-Type FLT3 Acute Myeloid Leukemia. PLoS ONE.

[179] Cortes J., Perl A.E., Döhner H., Kantarjian H., Martinelli G., Kovacsovics T., Rousselot P., Steffen B., Dombret H., Estey E. (2018). Quizartinib, an FLT3 Inhibitor, as Monotherapy in Patients with Relapsed or Refractory Acute Myeloid Leukaemia: An Open-Label, Multicentre, Single-Arm, Phase 2 Trial. Lancet Oncol..

[180] Cloos J.J., Montesinos P., Fiedler W., Müller R., Krauter J., Sica S., Westermann J., Heidinga M.E., Ngai L.L., Oussoren-Brockhoff Y.J.M. (2021). Midostaurin in Patients (Pts) with Newly Diagnosed *FLT3*-Mutation Negative Acute Myeloid Leukemia (AML): Final Results and Measurable Residual Disease (MRD) Analyses from the Unify Trial. Blood.

[181] Montesinos P., Rodriguez-Veiga R., Bergua Burgues J.M., Algarra J.L., Botella C., Rodriguez Arboli E., Bernal Del Castillo T., Tormo M., Calbacho M., Salamero O. (2024). Final Results of Quiwi: A Double Blinded, Randomized Pethema Trial Comparing Standard Chemotherapy Plus Quizartinib Versus Placebo in Adult Patients with Newly Diagnosed *FLT3* -ITD Negative AML. Blood.

[182] Mosquera Orgueira A., Perez Encinas M., Pérez Míguez C., Rodriguez Veiga R., Bergua Burgues J.M., Lorenzo Algarra J., Botella C., Perez Simon J.A., Bernal T., Tormo M. (2024). Enhanced Validation of the *FLT3* -like Gene Expression Signature As a Predictive Biomarker for Quizartinib Response in *FLT3-ITD* Negative Acute Myeloid Leukemia: Expanded Cohort and Extended Follow-up from the Pethema Quiwi Trial. Blood.

[183] Haddad F.G., Kantarjian H. (2024). Navigating the Management of Chronic Phase CML in the Era of Generic BCR::ABL1 Tyrosine Kinase Inhibitors. J. Natl. Compr. Cancer Netw..

[184] Norsworthy K.J., Mulkey F., Scott E.C., Ward A.F., Przepiorka D., Charlab R., Dorff S.E., Deisseroth A., Kazandjian D., Sridhara R. (2020). Differentiation Syndrome with Ivosidenib and Enasidenib Treatment in Patients with Relapsed or Refractory IDH-Mutated AML: A U.S. Food and Drug Administration Systematic Analysis. Clin. Cancer Res..

[185] Zeidner J.F. (2020). Differentiating the Differentiation Syndrome Associated with IDH Inhibitors in AML. Clin. Cancer Res..

[186] Alotaibi A.S., Yilmaz M., Kanagal-Shamanna R., Loghavi S., Kadia T.M., DiNardo C.D., Borthakur G., Konopleva M., Pierce S.A., Wang S.A. (2021). Patterns of Resistance Differ in Patients with Acute Myeloid Leukemia Treated with Type I versus Type II FLT3 Inhibitors. Blood Cancer Discov..

[187] Smith C.C., Levis M.J., Perl A.E., Hill J.E., Rosales M., Bahceci E. (2022). Molecular Profile of FLT3-Mutated Relapsed/Refractory Patients with AML in the Phase 3 ADMIRAL Study of Gilteritinib. Blood Adv..

[188] Lopez-Villar O., Garcia J.L., Sanchez-Guijo F.M., Robledo C., Villaron E.M., Hernández-Campo P., Lopez-Holgado N., Diez-Campelo M., Barbado M.V., Perez-Simon J.A. (2009). Both Expanded and Uncultured Mesenchymal Stem Cells from MDS Patients Are Genomically Abnormal, Showing a Specific Genetic Profile for the 5q− Syndrome. Leukemia.

[189] Blau O., Baldus C.D., Hofmann W.-K., Thiel G., Nolte F., Burmeister T., Türkmen S., Benlasfer O., Schümann E., Sindram A. (2011). Mesenchymal Stromal Cells of Myelodysplastic Syndrome and Acute Myeloid Leukemia Patients Have Distinct Genetic Abnormalities Compared with Leukemic Blasts. Blood.

[190] Balderman S.R., Li A.J., Hoffman C.M., Frisch B.J., Goodman A.N., LaMere M.W., Georger M.A., Evans A.G., Liesveld J.L., Becker M.W. (2016). Targeting of the Bone Marrow Microenvironment Improves Outcome in a Murine Model of Myelodysplastic Syndrome. Blood.

[191] Vijayanarayanan A., Shaw B.M., Gibbons K., Inamdar K.V., Kuriakose P., Menon M.P. (2022). The Need for Rapid Cytogenetics in the Era of Unique Therapies for Acute Myeloid Leukemia. Blood Adv..

[192] Burd A., Levine R.L., Ruppert A.S., Mims A.S., Borate U., Stein E.M., Patel P., Baer M.R., Stock W., Deininger M. (2020). Precision Medicine Treatment in Acute Myeloid Leukemia Using Prospective Genomic Profiling: Feasibility and Preliminary Efficacy of the Beat AML Master Trial. Nat. Med..

[193] Duong V.H., Ruppert A.S., Mims A.S., Borate U., Stein E.M., Baer M.R., Stock W., Kovacsovics T., Blum W., Arellano M.L. (2023). Entospletinib with Decitabine in Acute Myeloid Leukemia with Mutant TP53 or Complex Karyotype: A Phase 2 Substudy of the Beat AML Master Trial. Cancer.

[194] Little R.F., Othus M., Assouline S., Ansher S., Atallah E.L., Lindsley R.C., Freidlin B., Gore S.D., Harris L., Hourigan C.S. (2022). Umbrella Trial in Myeloid Malignancies: The Myelomatch National Clinical Trials Network Precision Medicine Initiative. Blood.

[195] Harris L.N., Blanke C.D., Erba H.P., Ford J.M., Gray R.J., LeBlanc M.L., Hu-Lieskovan S., Litzow M.R., Luger S.M., Meric-Bernstam F. (2023). The New NCI Precision Medicine Trials. Clin. Cancer Res..

[196] NCI’s myeloMATCH Precision Medicine Initiative Pushes the Envelope in Myeloid Leukemia Care. https://sohoinsider.com/leukemia/ncis-myelomatch-precision-medicine-initiative-pushes-the-envelope-in-myeloid-leukemia-care/?v=1736392324.

[197] Snijder B., Vladimer G.I., Krall N., Miura K., Schmolke A.-S., Kornauth C., de la Fuente O.L., Choi H.-S., van der Kouwe E., Gültekin S. (2017). Image-Based Ex-Vivo Drug Screening for Patients with Aggressive Haematological Malignancies: Interim Results from a Single-Arm, Open-Label, Pilot Study. Lancet Haematol..

[198] Kornauth C., Pemovska T., Vladimer G.I., Bayer G., Bergmann M., Eder S., Eichner R., Erl M., Esterbauer H., Exner R. (2022). Functional Precision Medicine Provides Clinical Benefit in Advanced Aggressive Hematologic Cancers and Identifies Exceptional Responders. Cancer Discov..

[199] Liebers N., Bruch P.-M., Terzer T., Hernandez-Hernandez M., Paramasivam N., Fitzgerald D., Altmann H., Roider T., Kolb C., Knoll M. (2023). Ex Vivo Drug Response Profiling for Response and Outcome Prediction in Hematologic Malignancies: The Prospective Non-Interventional SMARTrial. Nat. Cancer.

[200] Schmid J.A., Festl Y., Severin Y., Bacher U., Kronig M.-N., Snijder B., Pabst T. (2023). Efficacy and Feasibility of Pharmacoscopy-Guided Treatment for Acute Myeloid Leukemia Patients Who Have Exhausted All Registered Therapeutic Options. Haematologica.

